# Inhibiting and protective factors of exclusive breastfeeding in an Island population in Spain: a longitudinal study

**DOI:** 10.1186/s13006-025-00800-x

**Published:** 2025-12-24

**Authors:** Seila Llorente-Pulido, Estefanía Custodio, Laura Otero-García

**Affiliations:** 1 Department of Preventive Medicine, Public Health and Microbiology, PhD Programme in Epidemiology and Public Health, Universidad Autónoma de Madrid, Madrid, Spain; 2 National Center for Tropical Medicine, Instituto de Salud Carlos III (ISCIII), Madrid, 28029 Spain; 3 CIBER Infectious Diseases, Instituto de Salud Carlos III (ISCIII), Madrid, 28029 Spain; 4 National School of Public Health, Instituto de Salud Carlos III (ISCIII), Madrid, 28029 Spain; 5 CIBER of Epidemiology and Public Health, Instituto de Salud Carlos III (CIBERESP, ISCIII), Madrid, 28029 Spain

**Keywords:** Exclusive breastfeeding, Maternity service provision, Maternity care, Pacifier, Nipple pain, Perception of insufficient milk, Longitudinal study

## Abstract

**Background:**

Breastfeeding provides important health benefits for both mothers and infants. In Spain, the rate of exclusive breastfeeding (EBF) at 6 months is 47%, which remains below the 60% target proposed by the World Health Organization for 2030. The objective of this study was to identify the protective and inhibiting factors associated with EBF in a population of pregnant women in Tenerife (Canary Islands, Spain).

**Methods:**

We conducted a longitudinal, prospective study among women attending midwife consultations for pregnancy check-ups at a primary healthcare center between November 2018 and January 2021. We followed 83 women, collecting data at different time points during pregnancy and postpartum (hospital stay, 15 days, 1 month, 4 months, and 6 months). Descriptive statistics and univariable and multivariable logistic regression analyses were performed.

**Results:**

The inhibiting factors for EBF were pacifier use during the hospital stay, on EBF after birth (OR: 0.06, 95% CI: 0.02, 0.23), pacifier use at 15 days (OR: 0.13, 95% CI: 0.04, 0.42) and at 4 months (OR: 0.23, 95% CI: 0.07, 0.79), gynecological problems of the woman (OR: 0.14, 95% CI: 0.03, 0.61), the presence of nipple pain at 15 days affecting EBF at 4 months (OR: 0.18, 95% CI: 0.06, 0.56) and at 6 months (OR: 0.21, 95% CI: 0.07, 0.68), perception of insufficient milk at 15 days (OR: 0.15, 95% CI: 0.05, 0.49) and at 1 month (OR: 0.06, 95% CI: 0.02, 0.20), and giving birth in a privately managed hospital without Baby-Friendly Hospital Initiative (BFHI) accreditation (OR: 0.17, 95% CI: 0.05, 0.66). Protective factors for EBF were the woman’s prior knowledge of the benefits of breastfeeding for her child (OR: 5.25, 95% CI: 1.03, 26.80) and for herself (OR: 3.98, 95% CI: 1.31, 12.02), as well as having a foreign nationality (OR: 3.04, 95% CI: 1.05, 8.80).

**Conclusions:**

The factors impacting EBF can be addressed with improved care practices for women during pregnancy, childbirth, and the early postpartum period. It is essential to train healthcare professionals and implement the BFHI in Spain, where it is primarily carried out in public hospitals, but not in private ones.

**Supplementary Information:**

The online version contains supplementary material available at 10.1186/s13006-025-00800-x.

## Background

Breastfeeding (BF) is recognized as the most cost-effective and efficient strategy for preventing maternal and infant morbidity and mortality [1, 2]. In particular, maintaining exclusive breastfeeding (EBF), defined as feeding the infant solely with breast milk, without any other food or liquid during the first six months of life, is associated with higher infant survival rates due to its unique and irreplaceable properties that promote optimal growth [3]. From a nutritional perspective, breast milk provides essential health benefits for infants by enhancing the absorption of key nutrients such as lipids and proteins, supporting the development of a balanced intestinal microbiota, and supplying bioactive components with anti-inflammatory and protective effects against various diseases [4, 5]. Moreover, certain components of human milk influence epigenetic programming, that is, the regulation of gene expression without altering DNA sequence, contributing to improved neurodevelopment and a reduced long-term risk of non-communicable diseases [6]. Direct breastfeeding also plays a crucial role in infant development, as it involves the coordinated movement of the tongue, jaw, and lips—essential for effective sucking and swallowing. This process supports adequate orofacial development, both skeletal and muscular, and is considered the first preventive measure against future dentofacial malocclusions [7]. In addition, breastfeeding on demand, which allows the infant to regulate both the frequency and duration of feedings, promotes appetite self-regulation and helps the baby develop a natural balance between hunger and satiety [8].

Accordingly, within the framework of its Global Nutrition Targets for 2025, the World Health Organization (WHO) urged Member States to increase the prevalence of exclusive breastfeeding—measured as the proportion of infants aged 0–5 months who were exclusively breastfed during the previous day—to at least 50%. Building on this progress, during the 78th World Health Assembly, WHO Member States adopted a resolution to raise this target to 60% and extend the deadline to 2030, as outlined in the *Global Nutrition Targets for 2030: Breastfeeding Report* [9, 10]. Despite global efforts, this target has not yet been achieved. According to the most recent data from WHO and UNICEF (2024) [11], the global prevalence of exclusive breastfeeding among infants aged 0–5 months is approximately 48%, while data for Europe remain limited. In Spain, the latest National Health Survey (2023) [12] estimates the prevalence of exclusive breastfeeding among infants under 6 months of age to be 47%. For the Canary Islands, available EBF data are reported for the period between 3 and 6 months and estimated at 60%. However, the measurement method is not specified, and the age range does not correspond to the WHO 0–5 months indicator; therefore, these data are not directly comparable and are not included in the comparison [13].

This suboptimal adherence to recommended EBF rates has been linked in the literature to a range of maternal, social and systemic factors. Maternal physical problems such as fatigue, breast pain, or cracked nipples [14], as well as low socioeconomic and cultural levels [15]. The lack of support from partners and close family members is also among the most significant barriers [16]. Additionally, the absence of support networks, early return to work, and insufficient public policies, economic, social, and health-related, have been identified as major challenges to initiating and maintaining EBF [17, 18]. The COVID-19 pandemic introduced further difficulties, as described by Loezar-Hernández et al. (2023), who explored midwives’ perceptions of breastfeeding support during that period [19].

To address these challenges, the WHO and UNICEF launched the *Baby-Friendly Hospital Initiative (BFHI)* in 1991. In Spain, the initiative is known as the *Initiative for the Humanization of Childbirth and Breastfeeding Care* (*iHAN*), which seeks to protect and promote maternal and child health by encouraging supportive policies, training healthcare professionals, and implementing measures to improve childbirth and breastfeeding practices [20, 21]. Although initiatives such as iHAN provide an important framework for supporting breastfeeding, the effectiveness of such programs in the Canary Islands remain unclear, and there is limited evidence regarding the specific factors influencing EBF in the region.

To date, no studies have examined these determinants comprehensively. Building on previous research exploring midwives’ perspectives on the barriers and facilitators to exclusive breastfeeding (EBF) in Tenerife [22, 23], the present study complements those findings by examining factors associated with exclusive breastfeeding from the mothers’ perspective. Specifically, it aims to identify both inhibiting and protective factors influencing EBF through the follow-up of mother-infant dyads during the first six months of life in a Basic Health Zone in Tenerife (Canary Islands, Spain).

## Methods

### Design, population and study area

This is a prospective longitudinal analytical observational study with repeated cross-sectional follow-up assessments, conducted on a sample of women attending their first midwife consultation at the San Isidro primary healthcare center in Tenerife (Canary Islands) for pregnancy follow-up between November 2018 and November 2019. This center is in one of the most populated municipalities in the southern part of Tenerife, characterized by its multicultural environment, facilitated by tourism and immigration from other Autonomous Communities of Spain, countries from the European Union, and primarily Latin America. The center belongs to the basic health zone of Granadilla, which includes 7 primary healthcare centers and serves 69,935 users. The San Isidro primary healthcare center provides care to 15,152 women of childbearing age of whom approximately 1,231 were pregnant during the study year (data provided by the Directorate of the Basic Health Zone of Granadilla).

Participants were followed prospectively until cessation of EBF, as the study aimed to identify factors associated with the continuation of EBF up to six months postpartum. When EBF cessation occurred, information on the timing and reasons for discontinuation was collected, and no further EBF-specific follow-up interviews were scheduled thereafter, in accordance with the study protocol.

A formal power analysis was not conducted prior to recruitment, as the study aimed to include as many eligible women as possible during a one-year period (November 2018 to November 2019). This pragmatic approach, based on consecutive sampling (eligible participants are enrolled as they present within a defined timeframe), is considered appropriate in real-world clinical observational studies to capture the natural flow of patients and reduce selection bias [24].

The study’s inclusion criteria were pregnant women attending their first pregnancy follow-up appointment with a midwife with a:

- Pregnancy up to 13 weeks and 6 days.
- Low-risk pregnancy.
- Desire for monthly pregnancy follow-up with a primary care midwife.
- Favourably progressing pregnancy.

The exclusion criteria were pregnant women attending their first midwife appointment with a:

- Pregnancy loss at any time during the pregnancy.
- Language barrier (defined as an inability to understand or communicate adequately in Spanish).

The recruitment period for participants ran from November 6, 2018, to November 20, 2019. The follow-up period extended until January 2021.

### Data collection and study variables

Interviews were conducted using self-designed questionnaires (see SI). The questionnaires were piloted three months before data collection: initial versions were tested with pregnant women and follow-up versions with mothers during postpartum check-ups, none of whom were eligible or recruited as participants in the study. In total, 18 questionnaires were piloted that were subsequently refined based on feedback received and lessons learned. They were structured, interviewer-administered quantitative questionnaires composed mainly of closed-ended items designed to collect standardized information on sociodemographic, clinical, and behavioral variables.

In the initial interview, information was collected on sociodemographic aspects (age, marital status, education, nationality, monthly family income), pregestational personal health history (chronic illness, medication use, substance use), anthropometric data (pregestational body mass index and weight gain during pregnancy), gynecological-obstetric history (gynecological problems, parity), and current pregnancy data (follow-up consultations attendance, attendance at maternity/paternity preparation classes). No information was obtained from medical records. Therefore, all variables were based on participants’ self-reported responses rather than clinical assessments.

In the interview conducted 15 days after childbirth, two questionnaires were used:

- Questionnaire on hospital stay, collecting data on the delivery (type and place of delivery, onset, use of analgesia, gestational age), the newborn (weight, sex, Apgar score, admission), and type of breastfeeding and care (skin-to-skin contact, defined as direct, immediate, and uninterrupted mother–infant contact for at least the first two hours after birth [25, 26]; use of pacifier, nipple shield, or bottle).
- 15-day questionnaire, collecting data on breastfeeding 15 days after delivery, presence of breast pain describing exact location (e.g. nipple) and intensity, care (co-sleeping, baby-carrying methods, breastfeeding behaviour), aspects of the women’s mental health (responsibilities, fears about the new situation), the Edinburgh Postnatal Depression Scale (EPDS) was used in its validated Spanish version to assess depressive symptoms [27], and the LATCH assessment tool was used in its validated Spanish version to evaluate breastfeeding effectiveness [28].

In the one-month interview, information is again collected on breastfeeding, care, mental health aspects, the Edinburgh Postnatal Depression Scale, and the LATCH assessment tool.

In the four-month interview, questions are asked about breastfeeding, care (use of pacifier, where the baby sleeps, whether complementary feeding was started), and the return to work (if the mother has returned to work, who cares for the baby in her absence, how she balances childcare and work).

**Fig. 1:**
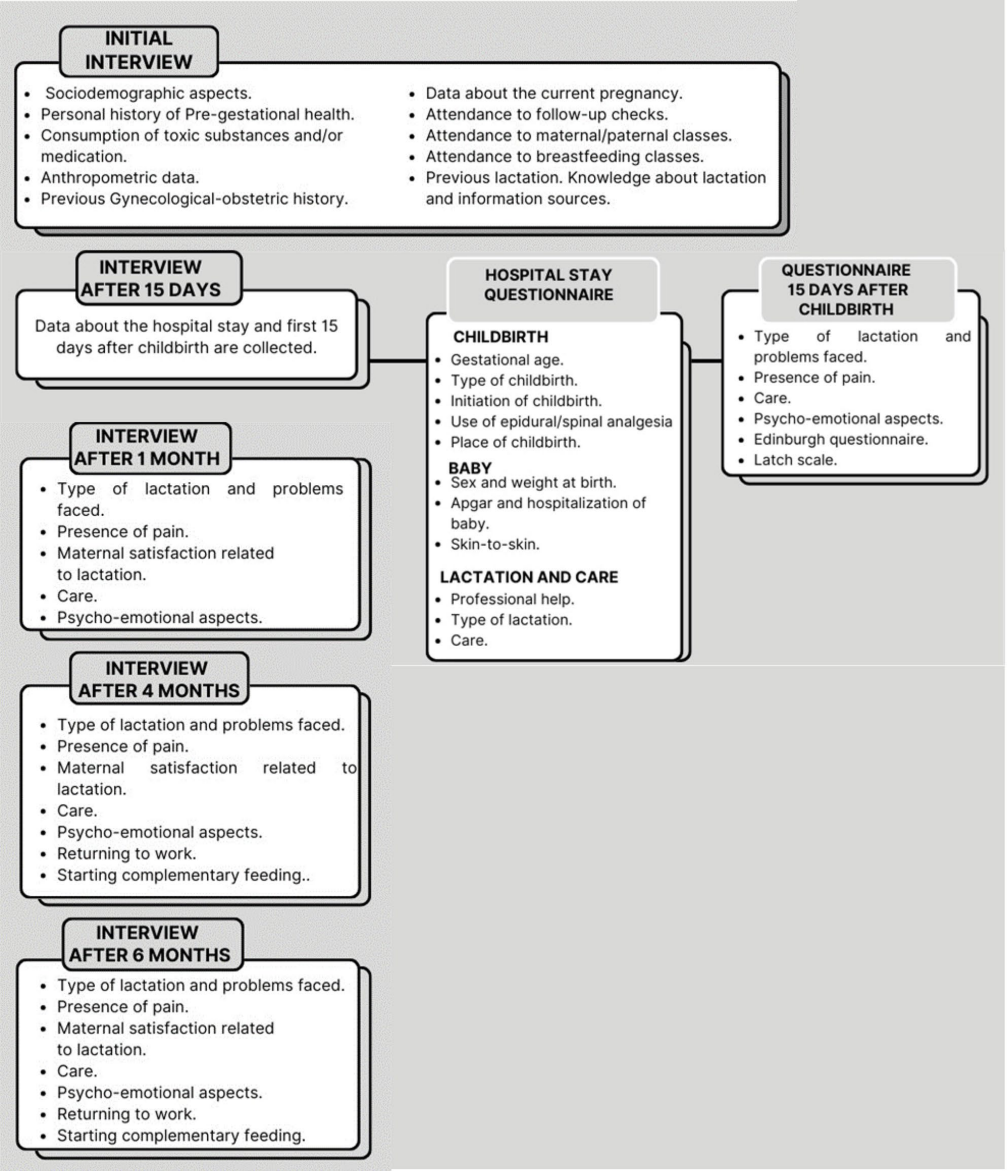
Information collected with questionnaires during the interviews at each study time point

In the six-month interview, information is collected once more on breastfeeding, care, return to work, and whether complementary feeding was started (Fig. 1 and S1).

Additionally, at the end of each follow-up questionnaire (15 days, one month, four months, and six months), an open-ended question was included to explore any difficulties the women experienced with breastfeeding. In case of breastfeeding discontinuation, the reason for stopping was asked. The responses to this question were combined into a limited number of categories (e.g. answers such as “the baby was not satisfied,” “I ran out of milk,” or “my breasts dried up” were grouped under the category “perception of insufficient milk”) and analyzed quantitatively. According to the literature, this concept refers to the mother’s perception of inadequate milk production based on her observation that the baby is not satisfied, remains hungry after feeding, or the appearance of her breasts (they see it as soft or they notice that milk does not flow or is watery) [29]. Therefore, although the questionnaire included one open-ended item, the responses were transformed into categorical variables for quantitative analysis. The study was thus conducted and analyzed within a quantitative analytical observational framework rather than as a mixed-methods design.

The dependent variable was exclusive breastfeeding (EBF) measured at the following points in time: during the hospital stay, 15 days after childbirth, at one month, four months, and six months of the baby’s life. EBF was considered when the woman reported that her child was fed exclusively with breast milk, without introducing any other food [18]. The questionnaire did not include follow-up questions to identify rare or occasional deviations from exclusivity; therefore, reports of EBF were treated as fully exclusive for the entire interval.

The initial questionnaire was completed in person with the midwife, the author of this work (SLP), during the pregnancy follow-up. The questionnaires regarding the hospital stay and at 15 days were collected in person during the postpartum consultation with the midwife at 15 days. The one month, four months, and six months interviews were conducted by phone.

### Data analysis

We performed descriptive statistics for the dependent and independent variables of the study.

Subsequently, we carried out five univariable logistic regressions, one for each of the study periods, using as dependent variable the EBF rate at the time of data collection (first days in the hospital after delivery, 15 days after birth, 1 month after birth, 4 months after birth and 6 months after birth).

We then performed the corresponding five multivariable logistic regressions including all variables that were significant at the *p* < 0.05 level in the univariable analyses and constructed the multivariable models with a forward stepwise approach, keeping in the models the variables that were significant at the *p* < 0.05 level. We applied the regressions using a penalized likelihood approach, the *Firth method*, to reduce the small-sample bias in the maximum likelihood estimations. We used Stata version 16.0 to perform the statistical analysis.

The study and manuscript preparation adhered to the Strengthening the Reporting of Observational Studies in Epidemiology (STROBE) guidelines for cross sectional studies.

## Results

**Fig. 2:**
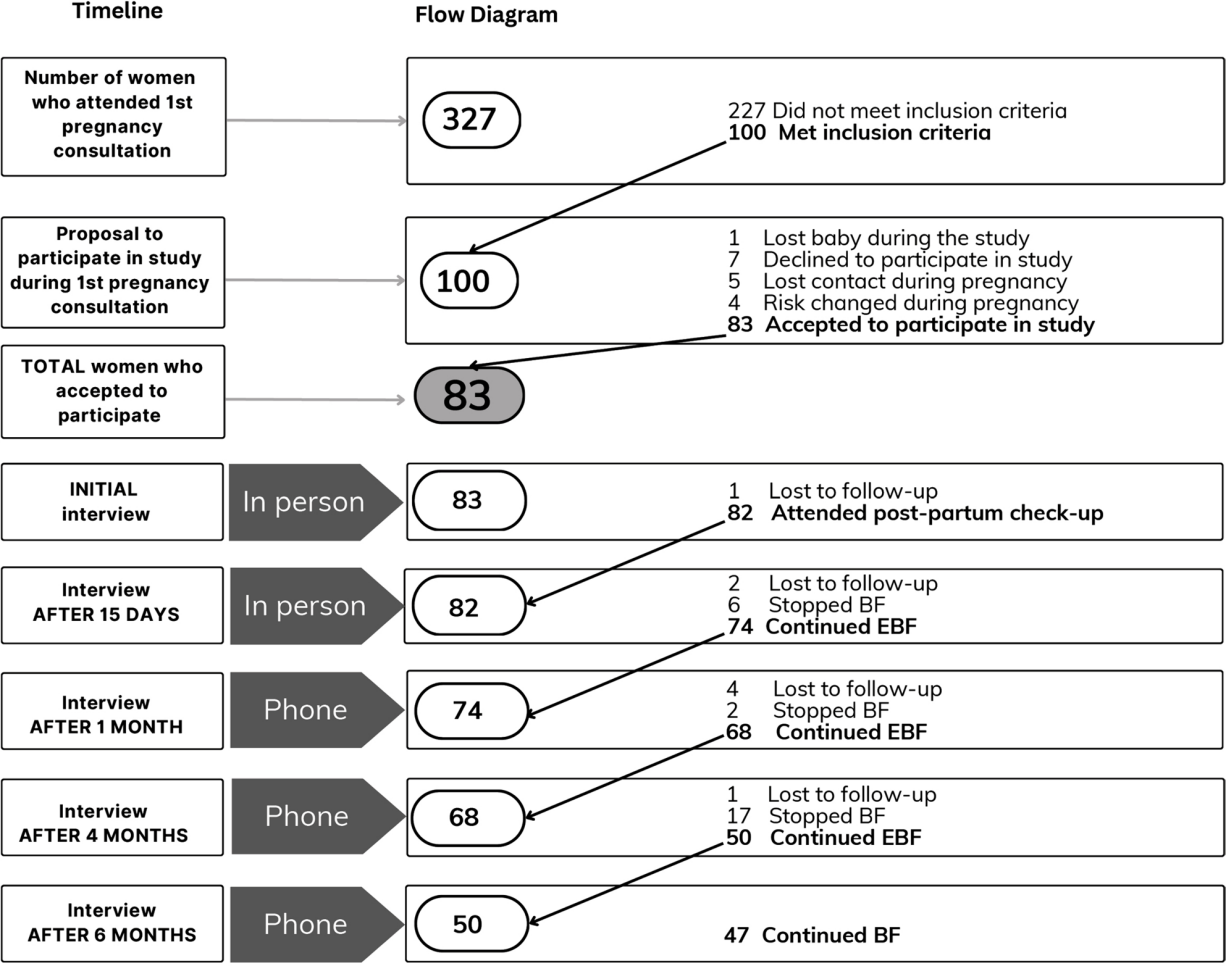
Flowchart of the number of women interviewed for breastfeeding follow-up at each study time point

A total of 327 pregnant women attended their first midwife consultation during the recruitment period. Of these, 100 met the study’s inclusion criteria. After receiving detailed information about the study, 83 women agreed to participate and were enrolled. The remaining 17 declined participation, or were excluded mainly due to pregnancy loss, or changes in clinical risk status. These 83 women comprised the final study sample. Figure 2 presents a flow diagram of participant recruitment, inclusion and follow-up throughout the study.

Per the study protocol, participants were followed prospectively until cessation of exclusive breastfeeding, as the study’s primary objective was to assess factors associated with the continuation of EBF up to six months postpartum. Therefore, the decreasing number of participants across follow-up points reflects both planned exit due to breastfeeding cessation and minor losses to follow-up (*n* = 8 in total: 1 before the 15-day interview, 2 before the 1-month interview, 4 before the 4-month interview, and 1 before the 6-month interview). No significant differences in baseline characteristics were observed between participants who completed the study and those lost to follow-up.

### General characteristics of the study population. (Table 1)

**Table 1:** General characteristics of the study population

Sociodemographic variables		*n* (%)
Maternal age	< 25 years 25–35 years > 35 years	16 (19.3) 52 (62.7) 15 (18.1)
Nationality	Spanish Foreign	47 (56.6) 36 (43.4)
Level of studies	Primary studies Vocational training Higher education	23 (27.7) 40 (48.2) 20 (24.1)
Family income	< 1000€/month 1000–2000€/month >2000€/month	10 (12) 42 (50.6) 31 (37.3)
**Obstetric-gynecological variable**		n (%)
Gynecological problems	Yes No	12 (14.5) 71 (85.5)
Weight gain during pregnancy (kg)	> 11,5 9–11,49 < 8,99	59 (72) 9 (11) 14 (17.1)
Number of children	First child Already has 1 or more	49 (59) 34 (41)
Type of childbirth	Eutocic Assisted C-section	63 (75.9) 9 (10.8) 11 (13.3)
Anesthesia use	Yes No	50 (60.2) 33 (39.8)
Initiation of childbirth	Spontaneous Induced	47 (56.6) 36 (43.4)
Gestational age at birth	Full-term Premature	79 (95.2) 4 (4.8)
Place of childbirth	Publicly managed hospital Privately managed hospital	60 (72.3) 23 (27.7
**Pregnancy and care variables**		n (%)
Pregnancy follow-up	Complete Incomplete	68(81.9) 15 (18.1)
Attendance to maternity/paternity classes	Complete Incomplete Never Cancelled due to pandemic	10 (12.1) 21 (25.3) 45 (54.2) 7(8.4)
Previous knowledge about BF benefits for the baby	Yes No	71 (87.8) 10 (12.2)
Previous knowledge about BF benefits for the mother	Yes No	54 (65.9) 28 (34.1)
Decision to BF prior to childbirth	Yes No	75 (90.4) 8 (9.6)
**Newborn (NB) variables**		n (%)
NB weight range	2,5 –4 kg <2,5 or > 4 kg	74(89.2) 9(10.8)
Sex of NB	Male Female	43 (51.8) 40 (48.2)
5 min Apgar	Good adaptation state Moderately depressed	77 (92.8) 6 (7.2)
Hospitalization in Neonatal care unit	Yes No	16 (19.3) 67 (80.7)

Regarding sociodemographic variables, most women surveyed (62.7%) were between 25 and 35 years old. 43.4% were foreigners, mainly from Latin America (75%), followed by other European countries (22.2%), and Africa (2.7%). A significant proportion (72.3%) had completed at least a middle or higher level of education. Additionally, 61.4% were employed outside the home, 96.4% had a partner, and the majority reported a monthly family income above €1000 (87.9%).

In connection with the variables related to pregnancy follow-up and care, 81.9% of the women attended all their follow-up appointments with the midwife. On the other hand, 12.1% attended all maternity/paternity preparation classes. Despite this, 87.8% reported having prior knowledge about the benefits of breastfeeding for their baby, and 65.9% were aware of the benefits for themselves. The women indicated that their sources of information were their close circle (family and friends) and healthcare professionals. Furthermore, 90.4% women interviewed had already expressed a desire to breastfeed before giving birth.

Concerning variables related to pre-gestational health and obstetric-gynecological history, 8.4% women suffered from chronic illnesses such as migraine, stable pregestational hypothyroidism, asthma not requiring treatment, and celiac disease. Additionally, 14.5% reported having gynecological problems, with 9 women indicating they had polycystic ovary syndrome. 53% of women (*n* = 44) had a normal BMI prior to becoming pregnant, while 30.1% (*n* = 25) were overweight and 16.9% (*n* = 14) had class I obesity. With respect to substance use, 19 women (22.9%) were smokers before pregnancy, and 6 continued smoking during pregnancy (1–3 cigarettes per day), while 13 quit smoking.

Within fifteen days after childbirth, 24.4% women returned to a normal weight, while 75.6% were either overweight or obese. Regarding tobacco use, 3 women resumed smoking after childbirth. The Edinburgh Postnatal Depression Scale was administered at the 15-day interview, with 19.3% scoring at a level indicating risk of postpartum depression, which decreased to 8.1% when administered after 1 month.

As for newborn data, early contact was performed in 88% cases, with 75% being skin-to-skin contact with their mother.

Concerning EBF follow-up variables (see Table 2), the use of pacifiers increased from 25.6% during the hospital stay to 60.8% at one month, after which its usage started to decline (42% at six months). Regarding the presence of nipple pain, the highest incidence was observed at 15 days postpartum (29 cases, accounting for 34.9% women). Maternal perception of insufficient milk was the issue most reported by women at 15 days and at one month (26.5% and 37.8%, respectively).

**Table 2:** Characteristics follow-up variables

Follow-up variables		Hospital stay	At 15 days	After 1 month	After 4 months	After 6 months
		*n* (%)	*n* (%)	*n* (%)	*n* (%)	*n* (%)
Use of pacifier	Yes No	21 (25.6) 61 (74.4)	38 (46.3) 44 (53.7)	45(60.8) 29 (39.2)	37(54.4) 31 (45.6)	21 (42) 29 (58)
Nipple pain	Yes No		29 (34.9) 54 (65.1)	11 (14.9) 63(85.1)	7(9) 61(91)	2(4) 48(96)
Maternal perception of insufficient milk	Yes No		22(26.5) 61(73.5)	28(37.8) 46(62.2)	1(1.5) 67(98.5)	3(6) 47(94)
Maternal satisfaction with breastfeeding	Very satisfied Satisfied A little satisfied Unsatisfied			48(65.8) 19(26) 6(8.2) 0	42(61.8) 15(22) 7(10.3) 4(5.9)	40(80) 9(18) 1(2) 0
Questioning if she is a good mother	Yes No		57 (67.9) 26 (32.1)	48(64.9) 26 (35.1)		
Is she anxious or afraid of the new responsibilities	Yes No		56 (66.7) 27 (33.3)	40 (54.1) 34 (45.9)		
Starting complementary feeding	Yes No				5 (7.3) 63 (92.6)	46 (92) 4 (8)
Returning to work	Yes No				6 (8.8) 62 (91.2)	10 (20) 40 (80)

Regarding the variables assessed in subsequent months, 7.3% of the women (5 of 68) reported initiating complementary feeding at four months, while the majority (92%; 46 of 50) did so at six months. In terms of return to work, 8.8% of the women who were still breastfeeding at four months and 20% of those breastfeeding at six months reported having resumed employment, regardless of whether they had been employed before pregnancy.

The percentage of women exclusively breastfeeding during the hospital stay was 67.1%, 59.8% at 15 days, 54.2% at one month, 44.6% at four months, and 39.8% at six months.

### Factors associated with EBF at different time periods

Table 3 shows the univariate analysis. The use of a pacifier during the hospital stay was significantly associated with a lower prevalence of EBF at all study time points, except the last one at 6 months. Similarly, the use of a pacifier during the first 15 days of life was associated with a lower EBF prevalence at 1 month, 4 months, and 6 months. Regarding the presence of nipple pain during the first 15 days, it was associated with lower EBF prevalence at 4 and 6 months. Maternal perception of insufficient milk was related to a lower EBF at 1 and 4 months. Having gynecological problems appeared as a factor associated with lower EBF rates during the hospital stay. The type of hospital where the delivery took place also showed a significant association with EBF, with women giving birth in private hospitals without iHAN (Baby-Friendly Hospital Initiative in Spain) accreditation being less likely to practice EBF at 4 and 6 months. On the other hand, a foreign nationality was associated with higher rates of EBF at 4 months. In addition, having prior knowledge about the benefits of breastfeeding for the baby was associated with higher EBF rates during the hospital stay, and at 4 months if the benefits for the mother are also known.

**Table 3:** Univariable analysis of the factors influencing EBF in each of the study periods

Covariates		During hospital stay *n* (%)	After 15 days *n* (%)	After 1 month *n* (%)	After 4 months *n* (%)	After 6 months *n* (%)
Use of a pacifier in the hospital	No	50(8.2)	44(72.1)	37(60.7)	32(53.3)	27(44.3)
	Yes	5(23.8)	5(23.8)	7(33.3)	4(19)	5(23.8)
	*p*	**< 0.0001**	**< 0.0001**	**0.03**	**0.007**	0.097
Use of a pacifier in the first 15 days after birth	No	.	28(63.6)	28(63.6)	24(55.8)	22(50)
	Yes	.	21(55.3)	16(42.1)	12(31.6)	10(26.3)
	*p*	.	0.441	**0.051**	**0.028**	**0.028**
Nipple pain during the first 15 days after birth	No	.	31(57.4)	29(53.7)	29(54.7)	27(50)
	Yes	.	18(64.3)	16(55.2)	8(27.6)	6(20.7)
	p	.	0.547	0.898	**0.018**	**0.009**
Perception of insufficient milk during the first 15 days after birth	No	.	43(71.67)	41(80.39)	36(59.02)	32(68.09)
	Yes	.	6(27.27)	4(17.39)	1(14.29)	1(33.33)
	*p*	.	**0.001**	**< 0.0001**	**0.053**	0.246
Gynecological problems	No	50(71.4)	44(62.9)	41(57.7)	32(45.7)	29(40.8)
	Yes	5(41.7)	5(41.7)	4(33.3)	5(41.7)	4(33.3)
	*p*	**0.043**	0.167	0.116	0.795	0.623
Place of childbirth (hospital)	Publicly managed	40(67.8)	37(62.7)	36(60)	31(51.7)	30(50)
	Privately managed	15(65.2)	12(52.2)	9(39.1)	6(27.3)	3(13)
	*p*	0.823	0.382	0.088	**0.049**	**0.002**
Previous knowledge about the benefits of breastfeeding for the baby	No	4(40)	5(50)	6(60)	3(30)	3(30)
	Yes	51(70.8)	44(61.1)	38(52.8)	33(46.5)	33(46.5)
	*p*	**0.052**	0.502	0.658	0.326	0.326
Previous knowledge about the benefits of breastfeeding for the mother	No	18(64.3)	15(53.6)	12(42.9)	8(28.6)	8(28.6)
	Yes	37(68.5)	34(63)	32(59.3)	28(52.8)	24(44.4)
	*p*	0.699	0.411	0.158	**0.037**	0.162
Nationality of the mother	Spanish	28(60.9)	26(56.5)	23(48.9)	17(36.2)	15(31.9)
	Foreign	27(75)	23(63.9)	22(61.1)	20(57.1)	18(50)
	*p*	0.177	0.5	0.27	**0.059**	0.095

Other aspects analyzed, such as attending maternal/paternal education classes, hospital practices involving mother-child separation, maternal psycho-emotional aspects, and returning to work, were not significantly associated with EBF in any of the studied periods.

Multivariable analyses were subsequently performed to identify independent factors associated with EBF at each time point (Table 4).

**Table 4:** Multivariable analysis of the factors influencing EBF in each of the study periods

Covariates		During hospital stay		After 15 days		After 1 month		After 4 months		After 6 months	
		n (%)	OR (CI 95%)	n (%)	OR (CI 95%)	n (%)	OR (CI 95%)	n (%)	OR (CI 95%)	n (%)	OR (CI 95%)
Use of a pacifier in the hospital	No	50(81.97)	Ref.	44(72.13)	Ref.			32(53.33)	Ref.		
	Yes	5(23.81)	0.06(0.02, 0.23)	5(23.81)	0.13(0.04, 0.42)			4(19.05)	0.23(0.07, 0.79)		
	p		< 0.0001		0.001				0.02		
Use of a pacifier in the first 15 days after birth	No									22(50)	Ref.
	Yes									10(26.32)	0.31(0.11, 0.89)
	p										0.029
Nipple pain during the first 15 days after birth	No							29(54.72)	Ref.	27(50)	Ref.
	Yes							8(27.59)	0.18(0.06, 0.56)	6(20.69)	0.21(0.07, 0.68)
	p								0.003		0.01
Perception of insufficient milk during the first 15 days after birth	No			43(71.67)	Ref.	41(80.39)	Ref.				
	Yes			6(27.27)	0.15(0.05, 0.49)	4(17.39)	0.06(0.02, 0.2)				
	p				0.002		< 0.0001				
Gynecological problems	No	50(71.43)	Ref.								
	Yes	5(41.67)	0.14(0.03, 0.61)								
	p		< 0.001								
Place of childbirth (hospital)	Publicly managed									30(50)	Ref.
	Privately managed									3(13)	0.17(0.05, 0.66)
	p										0.01
Previous knowledge about the benefits of breastfeeding for the baby	No	4(40)	Ref.								
	Yes	51(70.83)	5.25(1.03, 26.80)								
	p		0.046								
Previous knowledge about the benefits of breastfeeding for the mother	No							8(28.47)	Ref.		
	Yes							28(52.83)	3.98(1.31, 12.02)		
									0.014		
Nationality of the mother	Spanish							17(36.17)	Ref.		
	Foreign							20(57.14)	3.04(1.05, 8.80)		
	p								0.04		

During the hospital stay, the use of a pacifier (OR: 0.06, 95% CI: 0.02, 0.23) and the presence of gynecological problems (OR: 0.14, 95% CI: 0.03, 0.61) were negatively associated with starting EBF but with small effect sizes. Conversely, having prior knowledge about the benefits of breastfeeding for the baby was positively associated with EBF (OR: 5.25, 95% CI: 1.03, 26.80).

In the first 15 days, the use of a pacifier during the hospital stay continued to be negatively associated with EBF (OR: 0.13, 95% CI: 0.04, 0.42). This was also true for maternal perception of insufficient milk (OR: 0.15, 95% CI: 0.05, 0.49), which was the only variable negatively associated with EBF at one month (OR: 0.06, 95% CI: 0.02, 0.20), as the negative association with the use of a pacifier lost significance during this period.

At four months, the variables negatively associated with EBF were the use of a pacifier during the hospital stay (OR: 0.23, 95% CI: 0.07, 0.79) and the presence of nipple pain during the first 15 days postpartum (OR: 0.18, 95% CI: 0.06, 0.56). On the other hand, mothers who had prior knowledge about the benefits of breastfeeding for themselves and those who were foreign were more likely to practice EBF, both with large effect sizes (OR: 3.98, 95% CI: 1.31, 12.02 and OR: 3.04, 95% CI: 1.05, 8.80, respectively). The use of a pacifier at 15 days, the perception of insufficient milk, and the place of delivery lost significance.

At six months, the variables negatively associated with EBF included the use of a pacifier (OR: 0.31, 95% CI: 0.11, 0.89), the presence of nipple pain during the first 15 days of life (OR: 0.21, 95% CI: 0.07, 0.68) and giving birth in a privately managed hospital without iHAN accreditation (OR: 0.17, 95% CI: 0.05, 0.66).

## Discussion

Our study identified several inhibitory factors for EBF, including the use of pacifiers (both during the hospital stay and at 15 days postpartum), nipple pain and maternal perception of insufficient milk within the first 15 days of the baby’s life, mother’s history of gynecological problems, and giving birth in a privately managed hospital without iHAN accreditation. Conversely, protective factors for EBF were the mother’s prior knowledge of the benefits of breastfeeding for herself and her baby, as well as having a foreign nationality.

Our study found that **pacifier** use during the hospital stay and the first 15 days postpartum was negatively associated with exclusive breastfeeding (EBF) at all follow-up points, emerging as one of the main inhibitory factors in our population. This result is consistent with other observational studies conducted in Spain and Brazil [18, 30], possibly because the pacifier replaces non-nutritive sucking at the breast, reducing feeding frequency and stimulation, which may interfere with milk production [31]. Evidence on this topic remains mixed. Buccini et al. (2016), in a systematic review including 46 studies, found that observational studies consistently reported an association between pacifier use and early EBF interruption, whereas randomized controlled trials (RCTs) did not, likely due to limited external validity and selective samples [30, 32–34]. The authors proposed three explanations: interference with breastfeeding dynamics, reverse causality related to early feeding difficulties, and greater adherence to breastfeeding-supportive practices among mothers who avoid pacifiers [30]. More recent meta-analyses, such as those by Tolppola et al. (2022) and Jaafar et al. (2016), found no significant effect of early pacifier use on EBF duration [32–38]. However, as noted by Kuitunen (2023), many of the RCTs included in these meta-analyses assessed ‘breast milk feeding’ rather than direct breastfeeding at the breast. As a result, they did not capture the potential impact of pacifier use on suckling patterns, latch, or maternal stimulation—key mechanisms through which pacifiers may influence breastfeeding success [39]. In line with Kuitunen, our recommendation is that pacifier use should be carefully weighed, considering possible benefits (e.g., prevention of Sudden Infant Death Syndrome) against potential risks (e.g., interference with EBF) [39].

Another significant factor negatively impacting EBF at 4 and 6 months was **nipple pain** during the first 15 days of the infant’s life. This finding is consistent with previous studies identifying nipple pain as a common reason for early discontinuation of breastfeeding [40, 41]. Breast pain, including nipple pain, can result from multiple causes (such as latch problems, tongue-tie, or infection), leading to breastfeeding difficulties [42]. Pain may also contribute to reduced milk production, as mothers experiencing discomfort may breastfeed less frequently, resulting in diminished breast stimulation and, consequently, lower milk supply [43]. Given these mechanisms, an adequate breastfeeding technique is essential for successful breastfeeding and for preventing pain-related complications. Therefore, healthcare professionals should provide early counseling and follow-up, observe the first feeds, correct positioning, and intervene when necessary to prevent early EBF discontinuation [44, 45].

Our results also reveal that maternal **perception of insufficient milk** is another inhibitory factor for EBF at 15 days and one month. This perception could coexist with or arise in contexts where other breastfeeding challenges are present, such as pacifier use or nipple pain. Although the direction of these relationships cannot be established in our study, the co-occurrence of breastfeeding challenges (such as nipple pain or pacifier use) and maternal perceptions of insufficient milk suggest that early difficulties may contribute to doubts about milk supply, which in turn can lead to discontinuation of EBF.

Understanding the physiology of breastfeeding and the baby’s behavior at the breast is crucial for EBF success. When the baby stimulates the breast by sucking, a hormonal response in the mother is triggered which allows the production of milk. This sucking can be nutritive, when the baby extracts milk for feeding purposes, or non-nutritive, playing a more emotional and self-regulatory or calming role [46]. Interference with breastfeeding—whether due to pain, suboptimal latch, or use of a pacifier—can potentially disrupt these processes and contribute to perceptions of insufficient milk.

The perception of insufficient milk has been identified in the literature as one of the main reasons for discontinuing EBF before 6 months [13, 47–49]. This may be due to a misinterpretation of the infant’s behavior and crying, but could also be due to cultural factors, beliefs, and inadequate knowledge about how breast milk production works [43, 50–51]. Because perceived insufficient milk is closely linked to low breastfeeding self-efficacy—defined as a mother’s confidence in her ability to initiate and sustain breastfeeding under various circumstances—interventions that strengthen maternal confidence are essential. Evidence shows that strategies aimed at improving breastfeeding self-efficacy, together with recommended hospital practices such as skin-to-skin contact, rooming-in, and breastfeeding counseling, can help prevent misperceptions about milk supply and support the continuation of EBF [52].

Our study indicates that giving **birth in a private hospital**, compared to a public one, decreased the likelihood of the child being exclusively breastfed at 6 months. Analyzing the characteristics of both hospitals, we see the public referral hospital in the study area is accredited with phase 2D of the iHAN certification, whereas no private hospital in Tenerife is currently in the process of accreditation. We believe these findings may be more related to current practices in these hospitals than to the type of hospital management. Von Seehausen et al. (2023) highlight that women who give birth in iHAN-accredited hospitals or in the process of accreditation are more likely to practice exclusive breastfeeding during their hospital stay, regardless of whether the hospital is publicly or privately managed [53]. According to the National Catalogue of Hospitals of Spain (2024), there are 765 hospitals (334 public and 431 private), although it does not specify how many provide maternity care [54]. The IHAN website reports 155 accredited hospitals but does not indicate their type of management [55]. After cross-checking each of these centers with the National Catalogue of Hospitals (2024), it was found that approximately 79% (122) of the accredited hospitals are publicly managed [54, 55]. Marinelli et al. (2019) compared exclusive breastfeeding (EBF) rates in hospitals with and without BFHI accreditation, identifying skin-to-skin contact and initiation of the first feed within the first half hour of life—both recommended by the initiative—as the most influential practices. Mothers who gave birth in accredited hospitals were more likely to implement these practices, thereby facilitating the initiation of EBF [56]. The study also underscores the relevance of breastfeeding support in maternity wards and participation in prenatal classes, factors that strengthen an informed decision to breastfeed [56]. Accordingly, it can be inferred that women delivering in BFHI-accredited hospitals have a higher probability of practicing EBF, a circumstance more likely to occur in publicly managed than in privately managed hospitals.

Our study identified that in women who reported previously **knowing about the benefits of breastfeeding** for their children, exclusive breastfeeding rates at 15 days postpartum were positively influenced. Furthermore, if they knew the benefits of breastfeeding for themselves, this positively influenced exclusive breastfeeding rates at 4 months. The effect sizes of the knowledge variable was much larger than for the previously reviewed ones. We believe that understanding the benefits of breastfeeding can empower mothers to maintain breastfeeding for a longer period, as demonstrated by the study by Iglesias-Rosado et al. (2021) [57]. This finding is consistent with other research showing a positive association between breastfeeding knowledge and the intention or decision to breastfeed, as well as the breastfeeding duration [58].

On the other hand, regarding the **mother’s nationality**, the results obtained show that more foreign women were exclusively breastfeeding at 4 months compared to Spanish women (again, with a large effect size), which aligns with other studies carried out in different regions of Spain [29]. Blanco and Otero-García (2021), in their study on the barriers and facilitators of exclusive breastfeeding among Latin American immigrant women in Madrid (Spain), identified that popular and spiritual beliefs about breastfeeding in the women’s country of origin are crucial to reinforce exclusive breastfeeding [59]. Furthermore, the process of cultural transformation and access to healthcare resources received in the host country could help overcome other recommendations from the country of origin that differ from the WHO recommendations to achieve exclusive breastfeeding [57, 59–60].

Many of the results obtained in this research through surveys conducted with mothers coincide with and complement the information extracted from interviews with primary care midwives in Tenerife conducted previously by the authors [22, 23].

Finally, it should be noted that the present research took place during the COVID-19 pandemic. In March 2020, a state of alarm was declared in Spain. Government-imposed mandates required citizens to stay home to prevent coronavirus infection and limit its spread [61]. This led to the suspension of most economic activities, with only essential activities being allowed to continue (such as healthcare, food, civil protection, and security, etc.). Throughout the pandemic, various protective measures were implemented, which varied depending on the disease situation at national level [61]. This context has influenced some of the results obtained compared to those observed in previous research. For instance, the variable “return to work” after the end of the 16 weeks maternity leave. Returning to work is considered one of the most significant factors for discontinuing EBF [62]. However, this study did not identify a direct relationship between returning to work and discontinuation of EBF. This could be explained because most mothers who participated in this study did not physically return to their workplaces, either because teleworking had been implemented or because they were on a temporary layoff. Women highlighted that this situation allowed them to continue with EBF practices while staying at home [63]. This explanation is supported by the studies of Whyte et al. (2024) and Loezar-Hernández et al. (2023) on the social and healthcare support in pregnancy and postpartum care during the COVID-19 pandemic and its impact on EBF [19, 64].

## Limitations

Among the study’s limitations, a possible response bias should be noted, as the researcher collecting the study data had a dual role as the midwife accompanying the women during their journey and as a researcher, which may have led to responses given out of a desire to please. In addition, as all data were obtained from interviewer-administered self-reports and not from medical records, recall and self-report biases may have occurred, potentially affecting the accuracy of certain clinical or behavioral variables. Nevertheless, the use of structured interviews and standardized questions helped to reduce variability in data collection.

Also, the study design focused on mothers who continued exclusive breastfeeding (EBF), with follow-up discontinued after self-reported cessation. Although data were collected at the moment of cessation, the lack of subsequent follow-up for these participants limits the ability to compare post-cessation experiences and may introduce selection bias toward women who successfully continued EBF. This design reflects the study’s specific aim of identifying factors associated with the *continuation* of EBF rather than its discontinuation.

Additionally, the definition of EBF was not based on a 24-hour food intake record of the baby, as recommended by WHO and UNICEF [65] for population studies, but rather on a retrospective question covering the period between visits. This difference in measurement approach may affect the comparability of our EBF prevalence estimates with national or global data collected following WHO/UNICEF recommendations; however, it does not influence the internal validity of our analyses regarding factors associated with the continuation of EBF within this cohort.

Furthermore, the relatively small sample size (*N* = 83) may have contributed to the wide confidence intervals observed in some estimates, indicating lower precision and a degree of uncertainty in the strength of certain associations. Despite this limitation, the study’s prospective longitudinal design and the use of robust statistical techniques, such as the Firth method, helped to mitigate potential bias related to sparse data and to enhance the reliability of the findings within the context of the available sample.

## Conclusions

In summary, this study identifies several factors associated with the continuation or discontinuation of EBF during the first six months of life. The findings suggest that both hospital practices and early postnatal experiences significantly influence breastfeeding outcomes. Specifically, pacifier use during the hospital stay was associated with a lower likelihood of continuing EBF at four months, while mothers reporting nipple pain or perceived insufficient milk had a reduced probability of maintaining EBF at six and one month, respectively. Conversely, mothers who reported being aware of the benefits of breastfeeding for themselves and their infants were more likely to maintain exclusive breastfeeding at different time points.

These associations highlight the importance of providing mothers with early, evidence-based, and continuous professional support throughout the perinatal period. Healthcare providers should receive specific training in lactation management to offer consistent guidance, reassurance, and individualized care, particularly during the critical early postpartum phase [66].

Finally, these results underscore the relevance of implementing and strengthening the Baby-Friendly Hospital Initiative (BFHI/iHAN) in both public and private healthcare facilities across Spain, including the Canary Islands. Although derived from a local context, the insights gained from this study may contribute to shaping international strategies aimed at promoting exclusive breastfeeding and improving maternal and infant health outcomes globally.

## Supplementary Information

Below is the link to the electronic supplementary material.

Supplementary Material 1

## Data Availability

No datasets were generated or analysed during the current study.
