# Supplementary material for: Inhibiting and protective factors of exclusive breastfeeding in an Island population in Spain: a longitudinal study
Source: Int Breastfeed J. 2025 Dec 24;21:8. doi: 10.1186/s13006-025-00800-x (PMC12849537; doi:10.1186/s13006-025-00800-x)
Supplement: Supplementary file 1 — Supplementary Material 1 [file 13006_2025_800_MOESM1_ESM.pptx]

## Slide 1
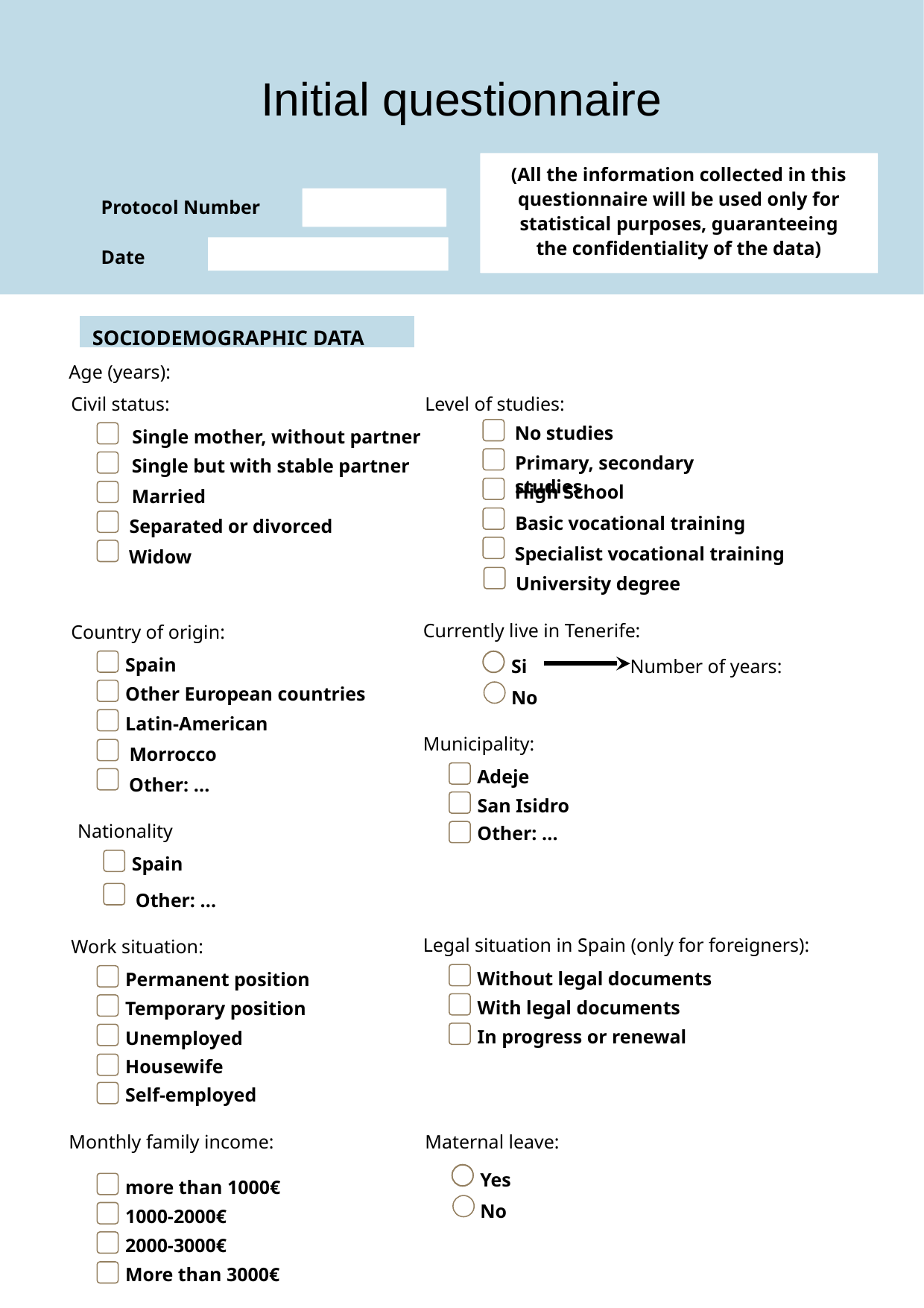

Initial questionnaire
(All the information collected in this questionnaire will be used only for statistical purposes, guaranteeing the confidentiality of the data)
Protocol Number
Date
SOCIODEMOGRAPHIC DATA
Age (years):
Civil status:
Level of studies:
No studies
Single mother, without partner
Primary, secondary studies
Single but with stable partner
High School
Married
Basic vocational training
Separated or divorced
Specialist vocational training
Widow
University degree
Currently live in Tenerife:
Country of origin:
Spain
Si
Number of years:
Other European countries
No
Latin-American
Municipality:
Morrocco
Adeje
Other: ...
San Isidro
Nationality
Other: ...
Spain
Other: ...
Legal situation in Spain (only for foreigners):
Work situation:
Without legal documents
Permanent position
With legal documents
Temporary position
In progress or renewal
Unemployed
Housewife
Self-employed
Monthly family income:
Maternal leave:
Yes
more than 1000€
No
1000-2000€
2000-3000€
More than 3000€

## Slide 2
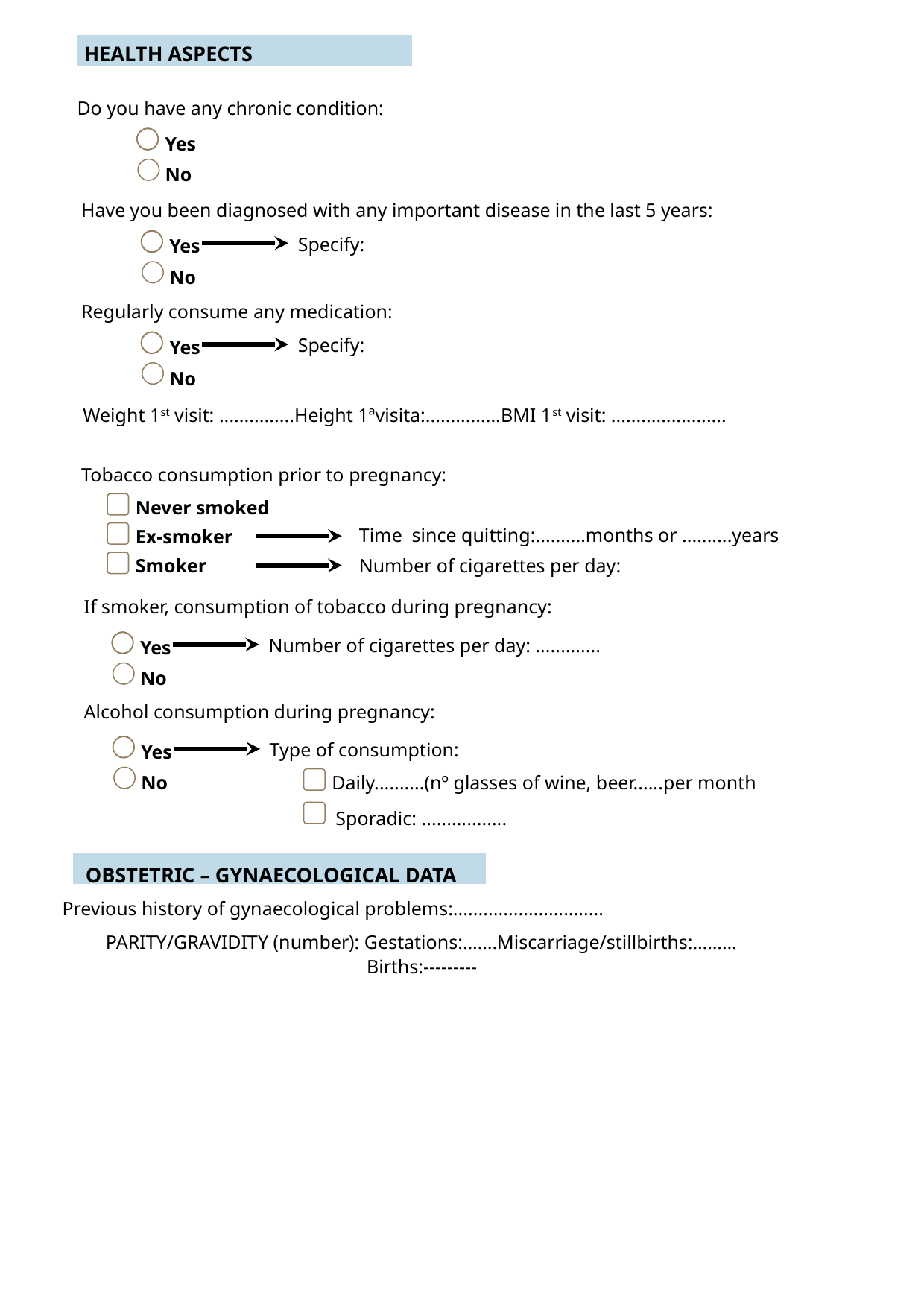

HEALTH ASPECTS
Do you have any chronic condition:
Yes
No
Have you been diagnosed with any important disease in the last 5 years:
Specify:
Yes
No
Regularly consume any medication:
Specify:
Yes
No
Weight 1st visit: ...............Height 1ªvisita:...............BMI 1st visit: .......................
Tobacco consumption prior to pregnancy:
Never smoked
Time since quitting:..........months or ..........years
Ex-smoker
Smoker
Number of cigarettes per day:
If smoker, consumption of tobacco during pregnancy:
Number of cigarettes per day: .............
Yes
No
Alcohol consumption during pregnancy:
Type of consumption:
Yes
Daily..........(nº glasses of wine, beer......per month
No
Sporadic: .................
OBSTETRIC – GYNAECOLOGICAL DATA
Previous history of gynaecological problems:..............................
PARITY/GRAVIDITY (number): Gestations:…….Miscarriage/stillbirths:………Births:---------

## Slide 3
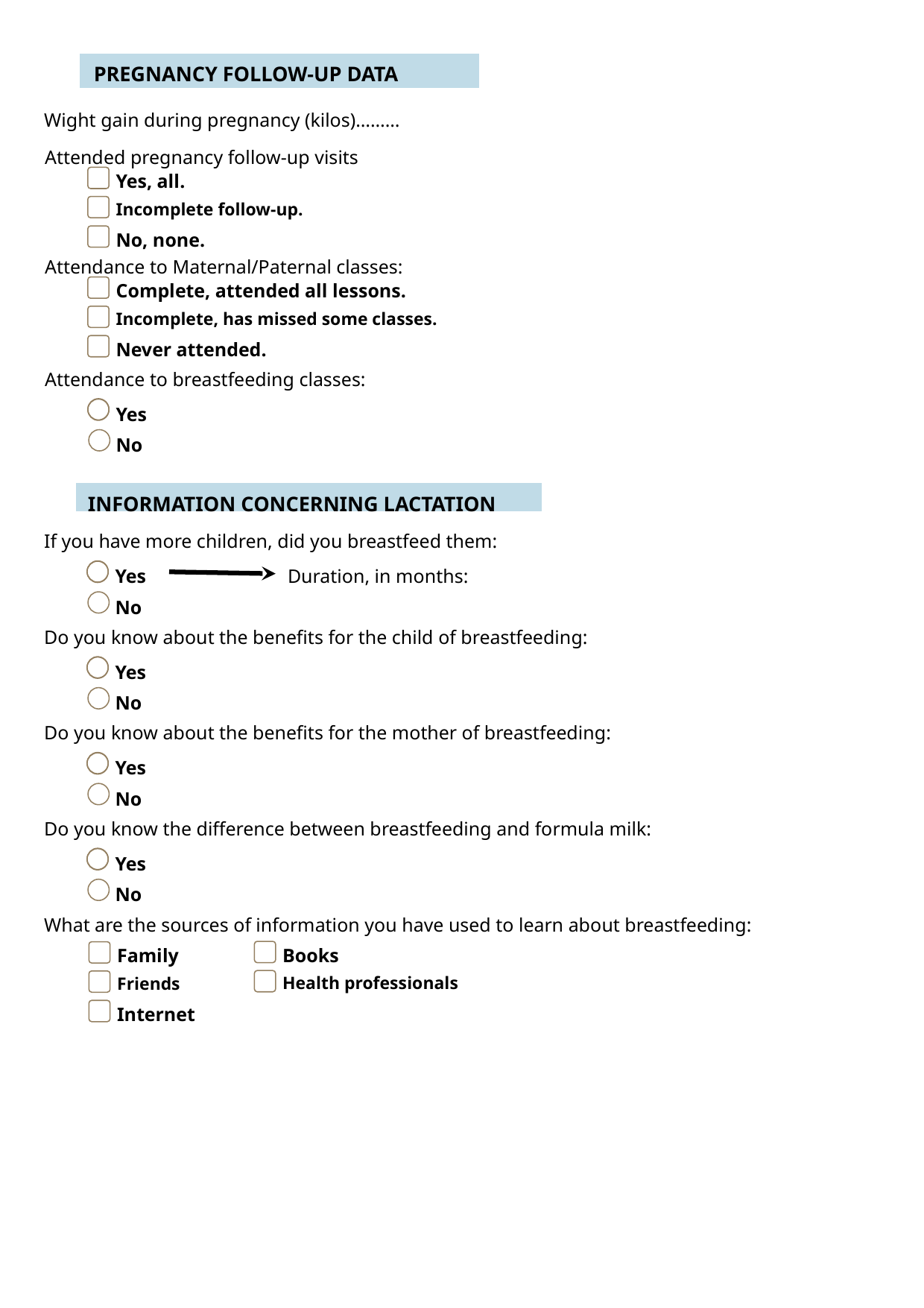

PREGNANCY FOLLOW-UP DATA
Wight gain during pregnancy (kilos)………
Attended pregnancy follow-up visits
Yes, all.
Incomplete follow-up.
No, none.
Attendance to Maternal/Paternal classes:
Complete, attended all lessons.
Incomplete, has missed some classes.
Never attended.
Attendance to breastfeeding classes:
Yes
No
INFORMATION CONCERNING LACTATION
If you have more children, did you breastfeed them:
Duration, in months:
Yes
No
Do you know about the benefits for the child of breastfeeding:
Yes
No
Do you know about the benefits for the mother of breastfeeding:
Yes
No
Do you know the difference between breastfeeding and formula milk:
Yes
No
What are the sources of information you have used to learn about breastfeeding:
Books
Family
Health professionals
Friends
Internet

## Slide 4
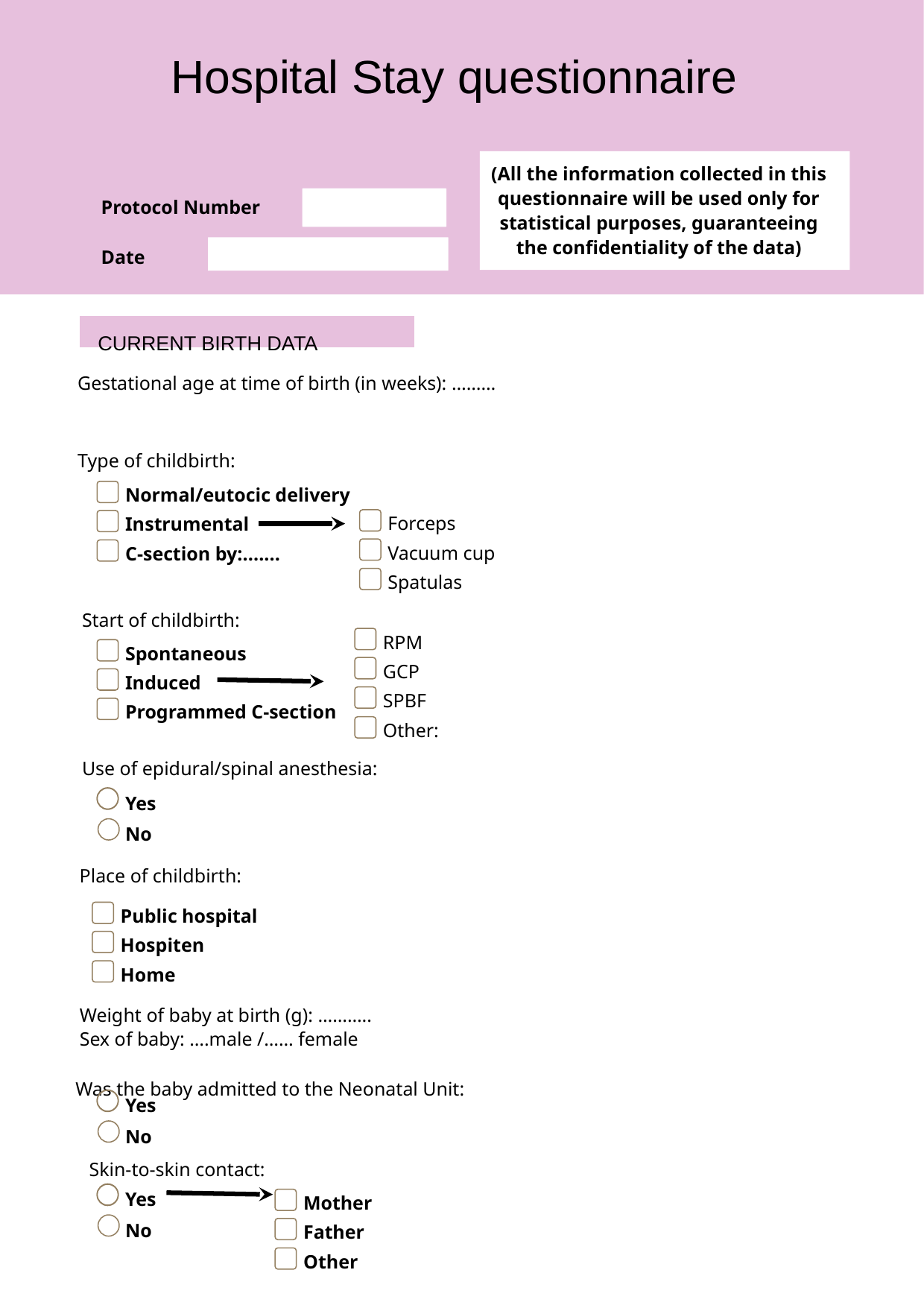

Hospital Stay questionnaire
(All the information collected in this questionnaire will be used only for statistical purposes, guaranteeing the confidentiality of the data)
Protocol Number
Date
 CURRENT BIRTH DATA
Gestational age at time of birth (in weeks): ………
Type of childbirth:
Normal/eutocic delivery
Forceps
Instrumental
Vacuum cup
C-section by:.......
Spatulas
Start of childbirth:
RPM
Spontaneous
GCP
Induced
SPBF
Programmed C-section
Other:
Use of epidural/spinal anesthesia:
Yes
No
Place of childbirth:
Public hospital
Hospiten
Home
Weight of baby at birth (g): ………..
Sex of baby: ….male /…… female
Was the baby admitted to the Neonatal Unit:
Yes
No
Skin-to-skin contact:
Yes
Mother
No
Father
Other

## Slide 5
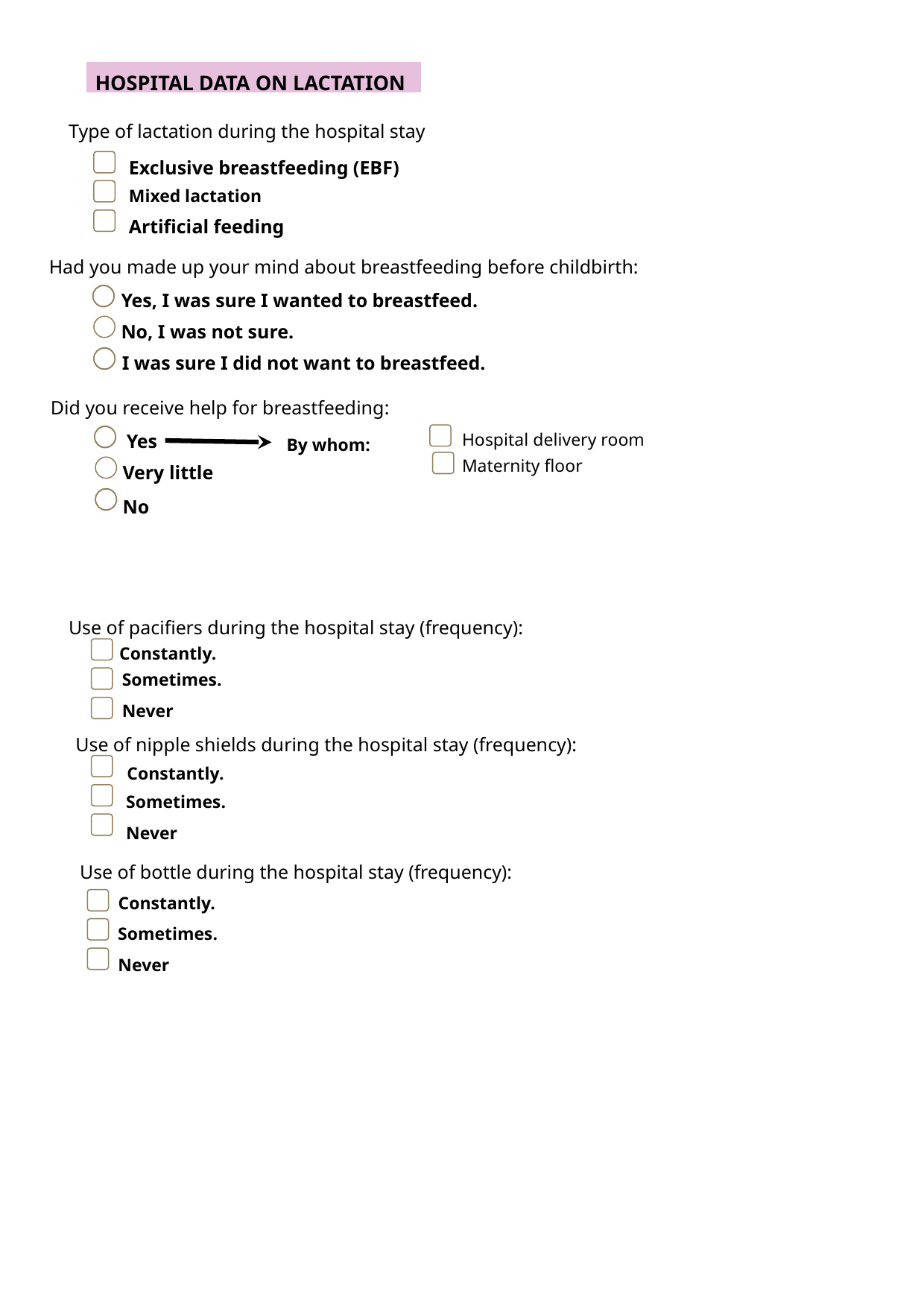

HOSPITAL DATA ON LACTATION
Type of lactation during the hospital stay
Exclusive breastfeeding (EBF)
Mixed lactation
Artificial feeding
Had you made up your mind about breastfeeding before childbirth:
Yes, I was sure I wanted to breastfeed.
No, I was not sure.
I was sure I did not want to breastfeed.
Did you receive help for breastfeeding:
Hospital delivery room
Yes
By whom:
Maternity floor
Very little
No
Use of pacifiers during the hospital stay (frequency):
Constantly.
Sometimes.
Never
Use of nipple shields during the hospital stay (frequency):
Constantly.
Sometimes.
Never
Use of bottle during the hospital stay (frequency):
Constantly.
Sometimes.
Never

## Slide 6
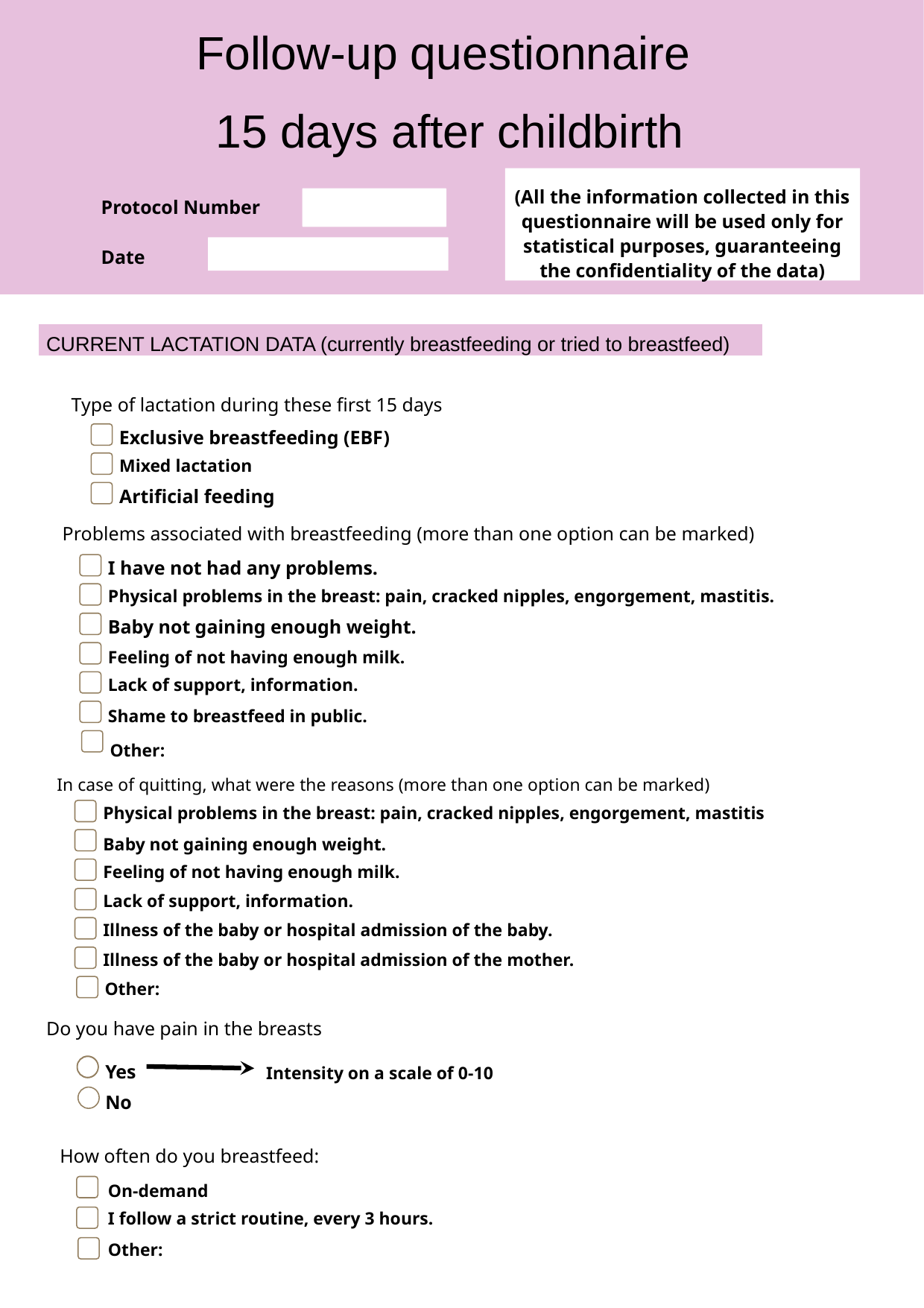

Follow-up questionnaire
15 days after childbirth
(All the information collected in this questionnaire will be used only for statistical purposes, guaranteeing the confidentiality of the data)
Protocol Number
Date
CURRENT LACTATION DATA (currently breastfeeding or tried to breastfeed)
Type of lactation during these first 15 days
Exclusive breastfeeding (EBF)
Mixed lactation
Artificial feeding
Problems associated with breastfeeding (more than one option can be marked)
I have not had any problems.
Physical problems in the breast: pain, cracked nipples, engorgement, mastitis.
Baby not gaining enough weight.
Feeling of not having enough milk.
Lack of support, information.
Shame to breastfeed in public.
Other:
In case of quitting, what were the reasons (more than one option can be marked)
Physical problems in the breast: pain, cracked nipples, engorgement, mastitis
Baby not gaining enough weight.
Feeling of not having enough milk.
Lack of support, information.
Illness of the baby or hospital admission of the baby.
Illness of the baby or hospital admission of the mother.
Other:
Do you have pain in the breasts
Yes
Intensity on a scale of 0-10
No
How often do you breastfeed:
On-demand
I follow a strict routine, every 3 hours.
Other:

## Slide 7
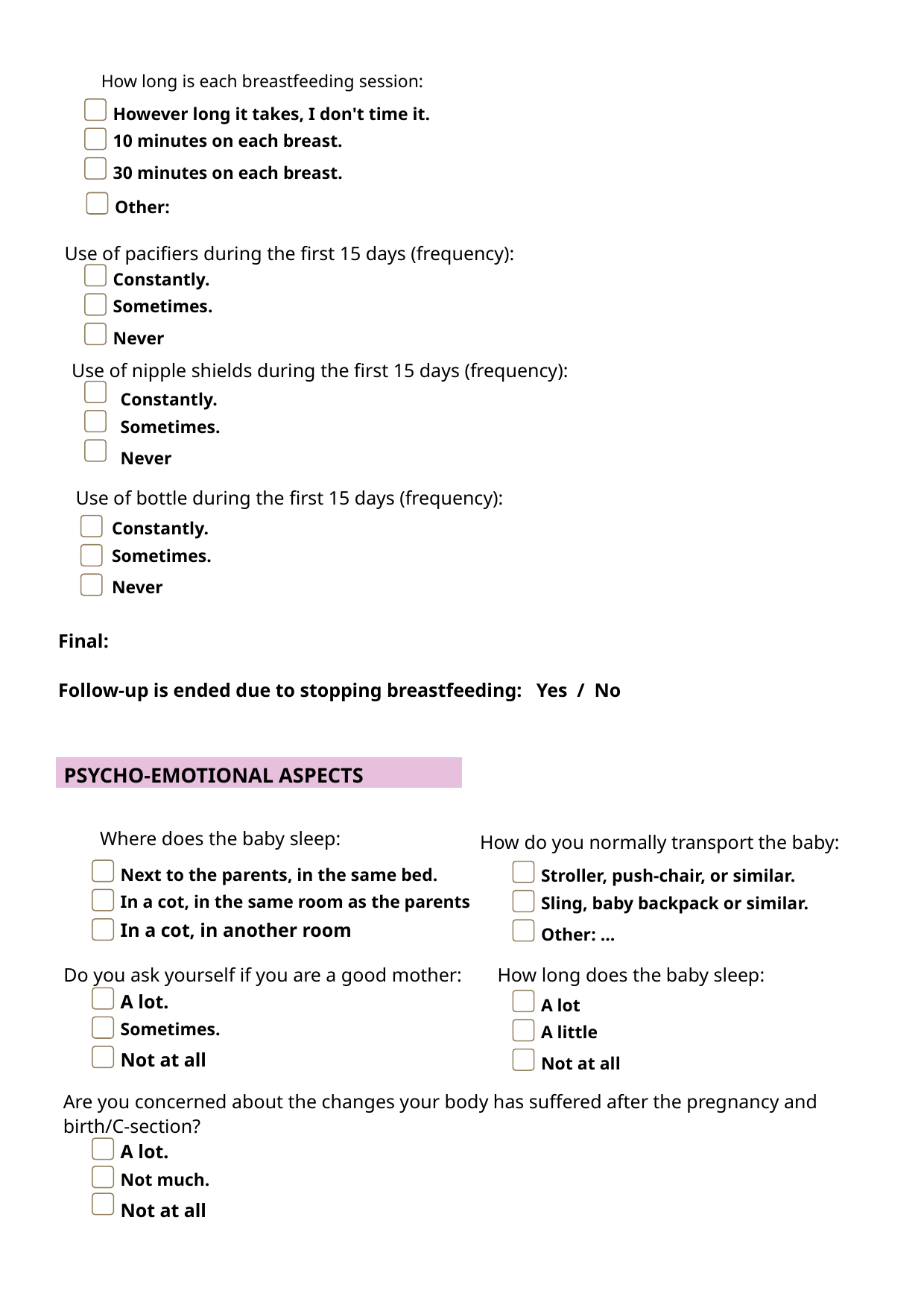

How long is each breastfeeding session:
However long it takes, I don't time it.
10 minutes on each breast.
30 minutes on each breast.
Other:
Use of pacifiers during the first 15 days (frequency):
Constantly.
Sometimes.
Never
Use of nipple shields during the first 15 days (frequency):
Constantly.
Sometimes.
Never
Use of bottle during the first 15 days (frequency):
Constantly.
Sometimes.
Never
Final:
Follow-up is ended due to stopping breastfeeding: Yes / No
PSYCHO-EMOTIONAL ASPECTS
 Where does the baby sleep:
How do you normally transport the baby:
Next to the parents, in the same bed.
Stroller, push-chair, or similar.
In a cot, in the same room as the parents
Sling, baby backpack or similar.
In a cot, in another room
Other: …
Do you ask yourself if you are a good mother:
 How long does the baby sleep:
A lot.
A lot
Sometimes.
A little
Not at all
Not at all
Are you concerned about the changes your body has suffered after the pregnancy and birth/C-section?
A lot.
Not much.
Not at all

## Slide 8
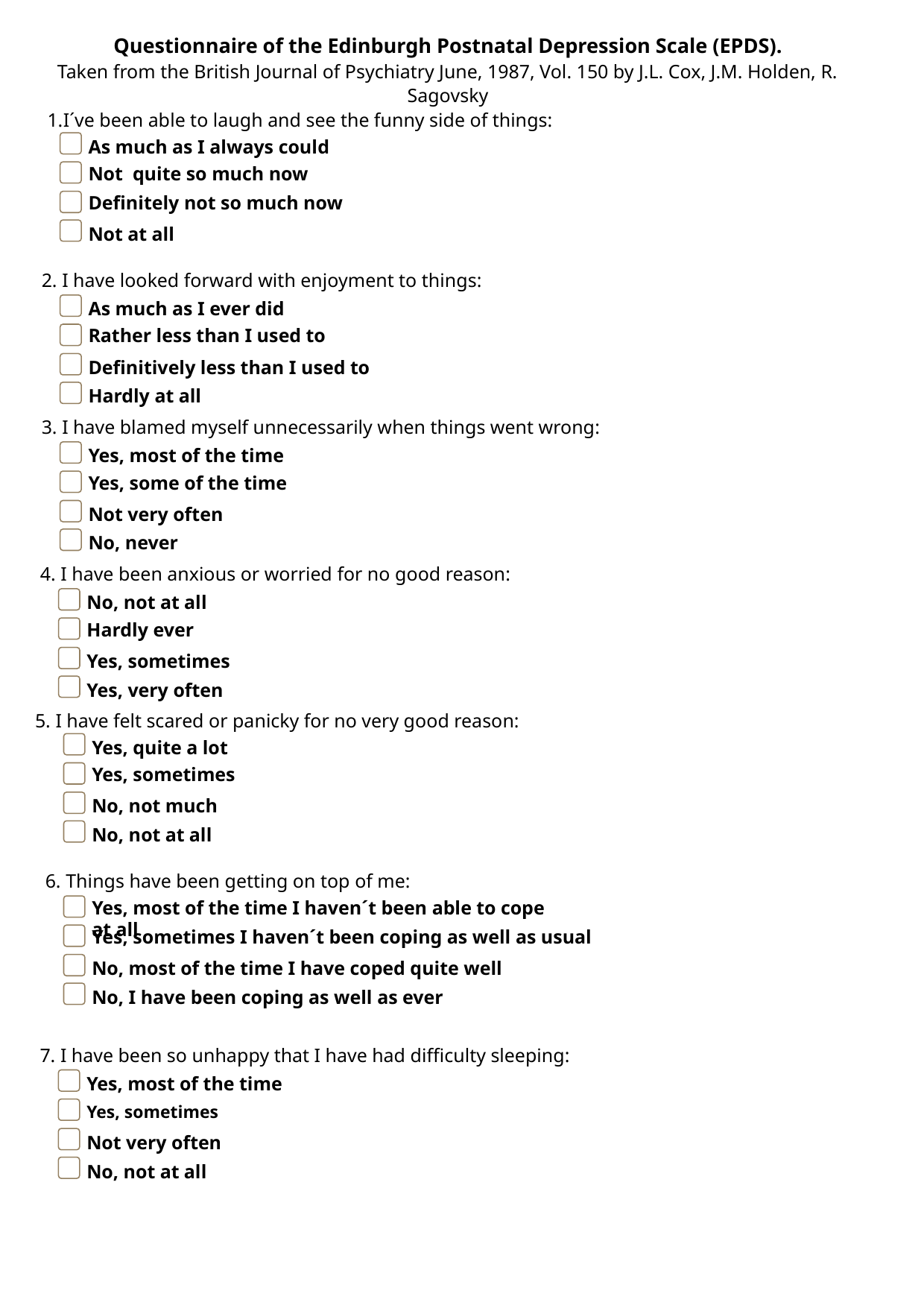

Questionnaire of the Edinburgh Postnatal Depression Scale (EPDS).
Taken from the British Journal of Psychiatry June, 1987, Vol. 150 by J.L. Cox, J.M. Holden, R. Sagovsky
I´ve been able to laugh and see the funny side of things:
As much as I always could
Not quite so much now
Definitely not so much now
Not at all
2. I have looked forward with enjoyment to things:
As much as I ever did
Rather less than I used to
Definitively less than I used to
Hardly at all
3. I have blamed myself unnecessarily when things went wrong:
Yes, most of the time
Yes, some of the time
Not very often
No, never
4. I have been anxious or worried for no good reason:
No, not at all
Hardly ever
Yes, sometimes
Yes, very often
5. I have felt scared or panicky for no very good reason:
Yes, quite a lot
Yes, sometimes
No, not much
No, not at all
6. Things have been getting on top of me:
Yes, most of the time I haven´t been able to cope at all
Yes, sometimes I haven´t been coping as well as usual
No, most of the time I have coped quite well
No, I have been coping as well as ever
7. I have been so unhappy that I have had difficulty sleeping:
Yes, most of the time
Yes, sometimes
Not very often
No, not at all

## Slide 9
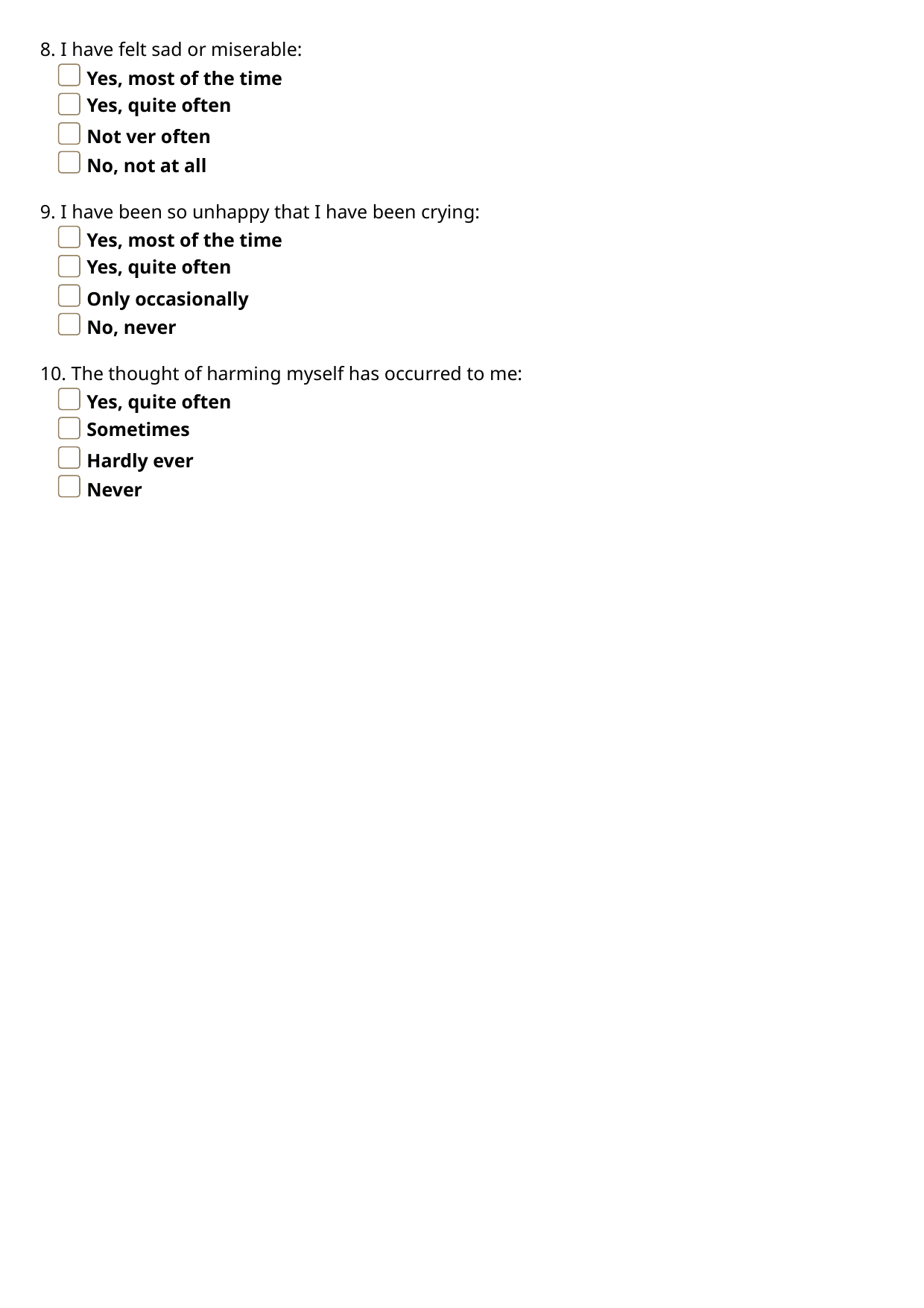

8. I have felt sad or miserable:
Yes, most of the time
Yes, quite often
Not ver often
No, not at all
9. I have been so unhappy that I have been crying:
Yes, most of the time
Yes, quite often
Only occasionally
No, never
10. The thought of harming myself has occurred to me:
Yes, quite often
Sometimes
Hardly ever
Never

## Slide 10
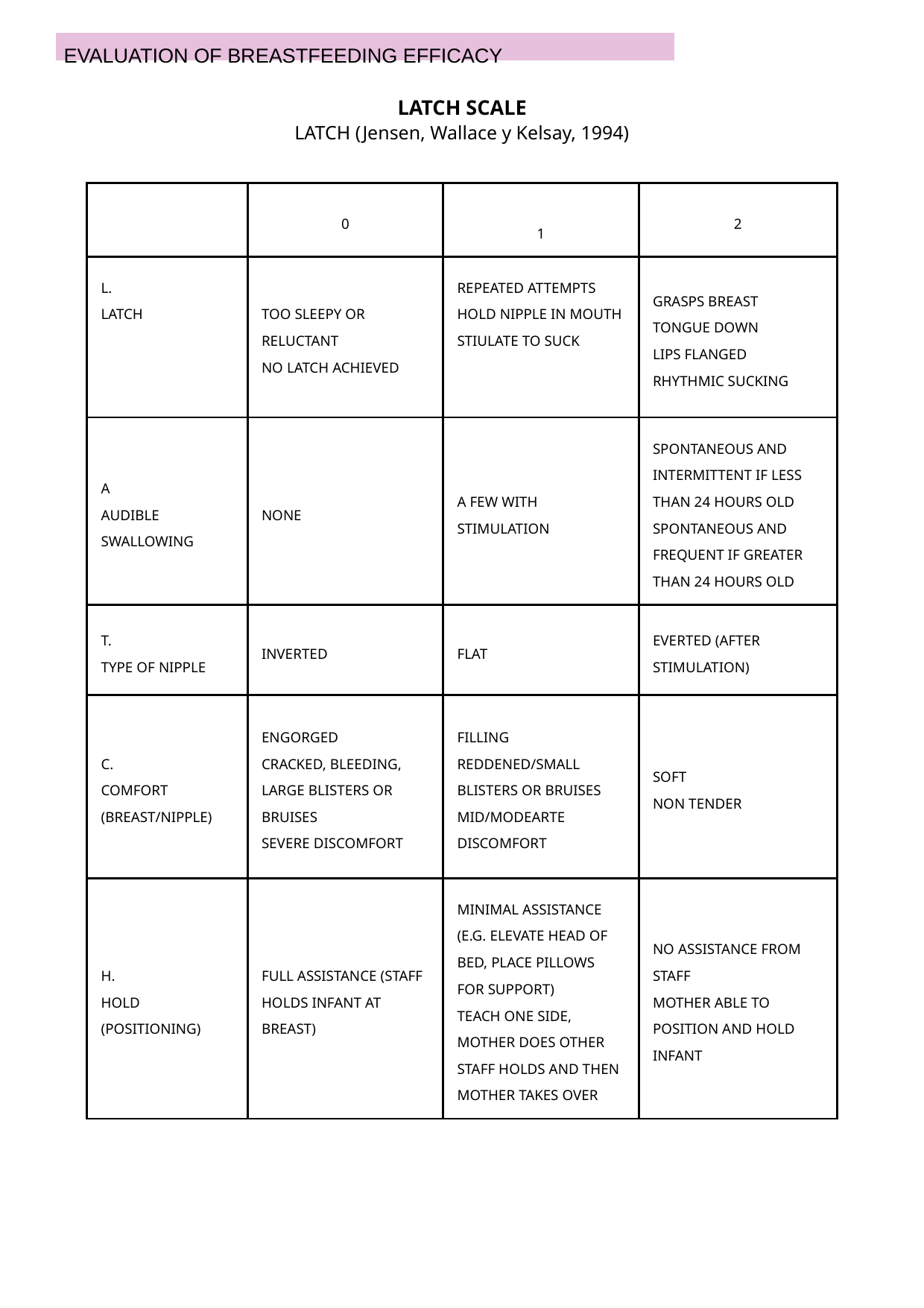

EVALUATION OF BREASTFEEDING EFFICACY
LATCH SCALE
LATCH (Jensen, Wallace y Kelsay, 1994)
| | 0 | 1 | 2 |
| --- | --- | --- | --- |
| L. LATCH | TOO SLEEPY OR RELUCTANT NO LATCH ACHIEVED | REPEATED ATTEMPTS HOLD NIPPLE IN MOUTH STIULATE TO SUCK | GRASPS BREAST TONGUE DOWN LIPS FLANGED RHYTHMIC SUCKING |
| A AUDIBLE SWALLOWING | NONE | A FEW WITH STIMULATION | SPONTANEOUS AND INTERMITTENT IF LESS THAN 24 HOURS OLD SPONTANEOUS AND FREQUENT IF GREATER THAN 24 HOURS OLD |
| T. TYPE OF NIPPLE | INVERTED | FLAT | EVERTED (AFTER STIMULATION) |
| C. COMFORT (BREAST/NIPPLE) | ENGORGED CRACKED, BLEEDING, LARGE BLISTERS OR BRUISES SEVERE DISCOMFORT | FILLING REDDENED/SMALL BLISTERS OR BRUISES MID/MODEARTE DISCOMFORT | SOFT NON TENDER |
| H. HOLD (POSITIONING) | FULL ASSISTANCE (STAFF HOLDS INFANT AT BREAST) | MINIMAL ASSISTANCE (E.G. ELEVATE HEAD OF BED, PLACE PILLOWS FOR SUPPORT) TEACH ONE SIDE, MOTHER DOES OTHER STAFF HOLDS AND THEN MOTHER TAKES OVER | NO ASSISTANCE FROM STAFF MOTHER ABLE TO POSITION AND HOLD INFANT |

## Slide 11
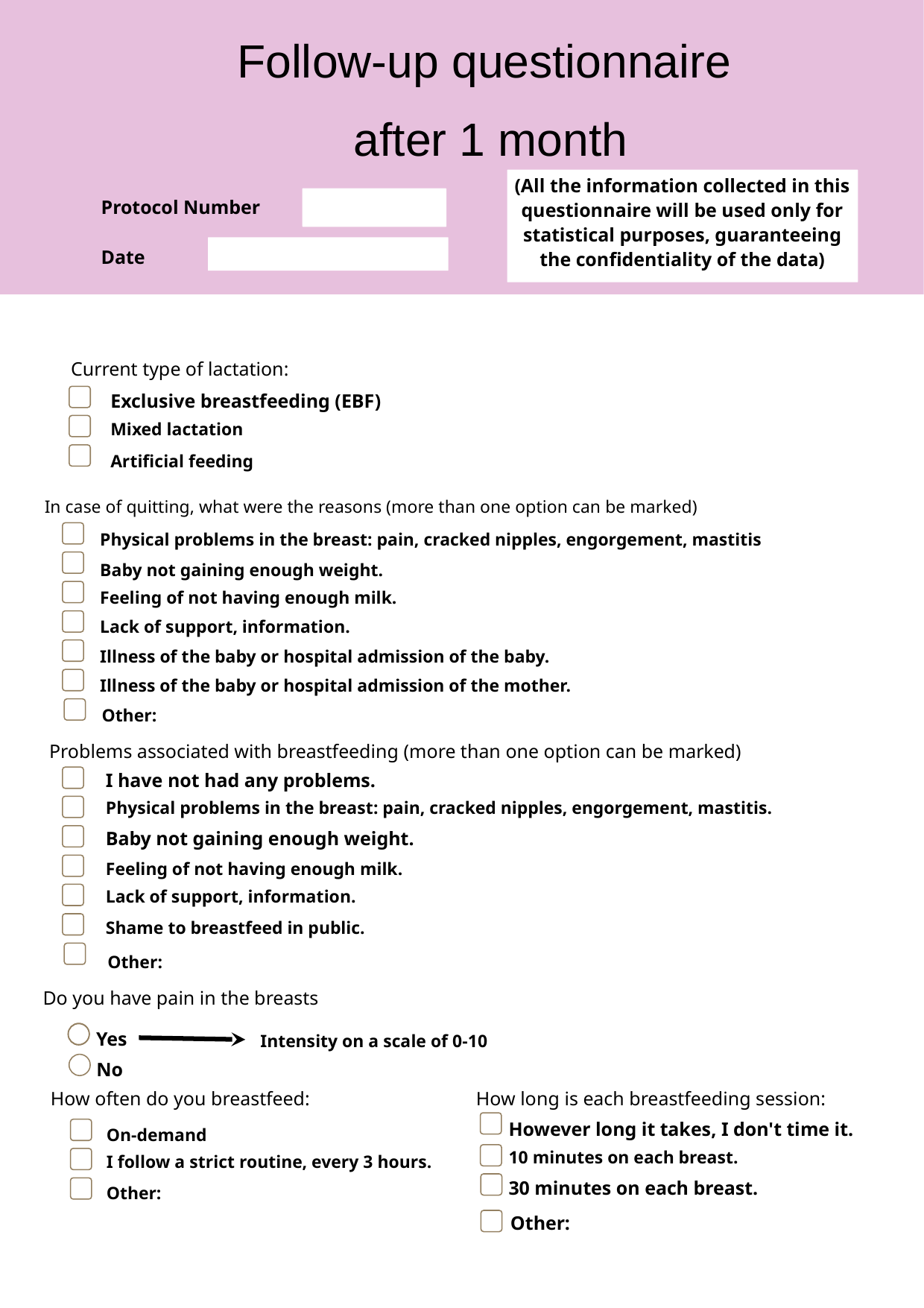

Follow-up questionnaire
after 1 month
(All the information collected in this questionnaire will be used only for statistical purposes, guaranteeing the confidentiality of the data)
Protocol Number
Date
Current type of lactation:
Exclusive breastfeeding (EBF)
Mixed lactation
Artificial feeding
In case of quitting, what were the reasons (more than one option can be marked)
Physical problems in the breast: pain, cracked nipples, engorgement, mastitis
Baby not gaining enough weight.
Feeling of not having enough milk.
Lack of support, information.
Illness of the baby or hospital admission of the baby.
Illness of the baby or hospital admission of the mother.
Other:
Problems associated with breastfeeding (more than one option can be marked)
I have not had any problems.
Physical problems in the breast: pain, cracked nipples, engorgement, mastitis.
Baby not gaining enough weight.
Feeling of not having enough milk.
Lack of support, information.
Shame to breastfeed in public.
Other:
Do you have pain in the breasts
Yes
Intensity on a scale of 0-10
No
How often do you breastfeed:
How long is each breastfeeding session:
However long it takes, I don't time it.
On-demand
10 minutes on each breast.
I follow a strict routine, every 3 hours.
30 minutes on each breast.
Other:
Other:

## Slide 12
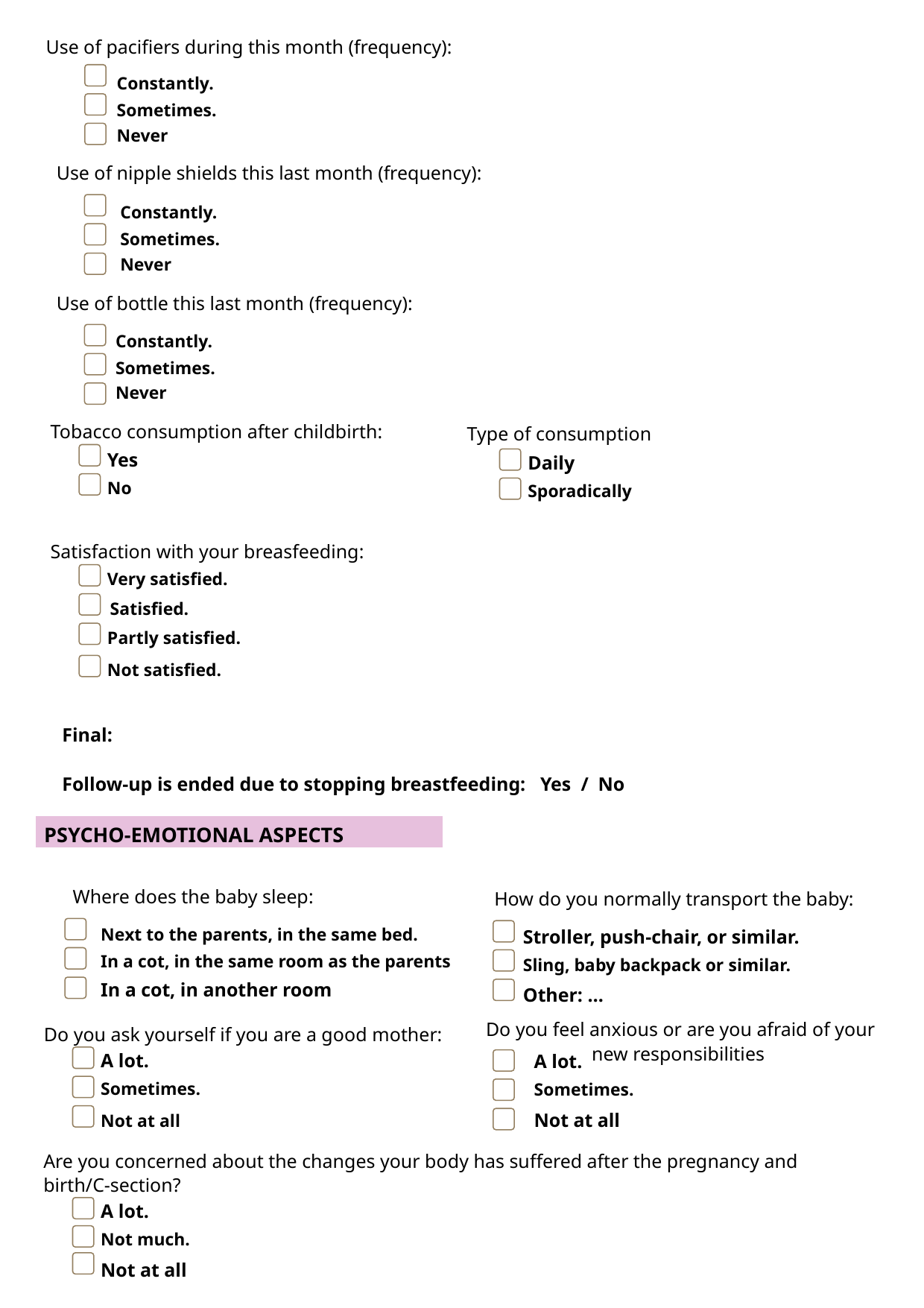

Use of pacifiers during this month (frequency):
Constantly.
Sometimes.
Never
Use of nipple shields this last month (frequency):
Constantly.
Sometimes.
Never
Use of bottle this last month (frequency):
Constantly.
Sometimes.
Never
Tobacco consumption after childbirth:
Type of consumption
Yes
Daily
No
Sporadically
Satisfaction with your breasfeeding:
Very satisfied.
Satisfied.
Partly satisfied.
Not satisfied.
Final:
Follow-up is ended due to stopping breastfeeding: Yes / No
PSYCHO-EMOTIONAL ASPECTS
ASPECTOS PSICOEMOCIONALES
 Where does the baby sleep:
How do you normally transport the baby:
Next to the parents, in the same bed.
Stroller, push-chair, or similar.
In a cot, in the same room as the parents
Sling, baby backpack or similar.
In a cot, in another room
Other: …
 Do you feel anxious or are you afraid of your new responsibilities
Do you ask yourself if you are a good mother:
A lot.
A lot.
Sometimes.
Sometimes.
Not at all
Not at all
Are you concerned about the changes your body has suffered after the pregnancy and birth/C-section?
A lot.
Not much.
Not at all

## Slide 13
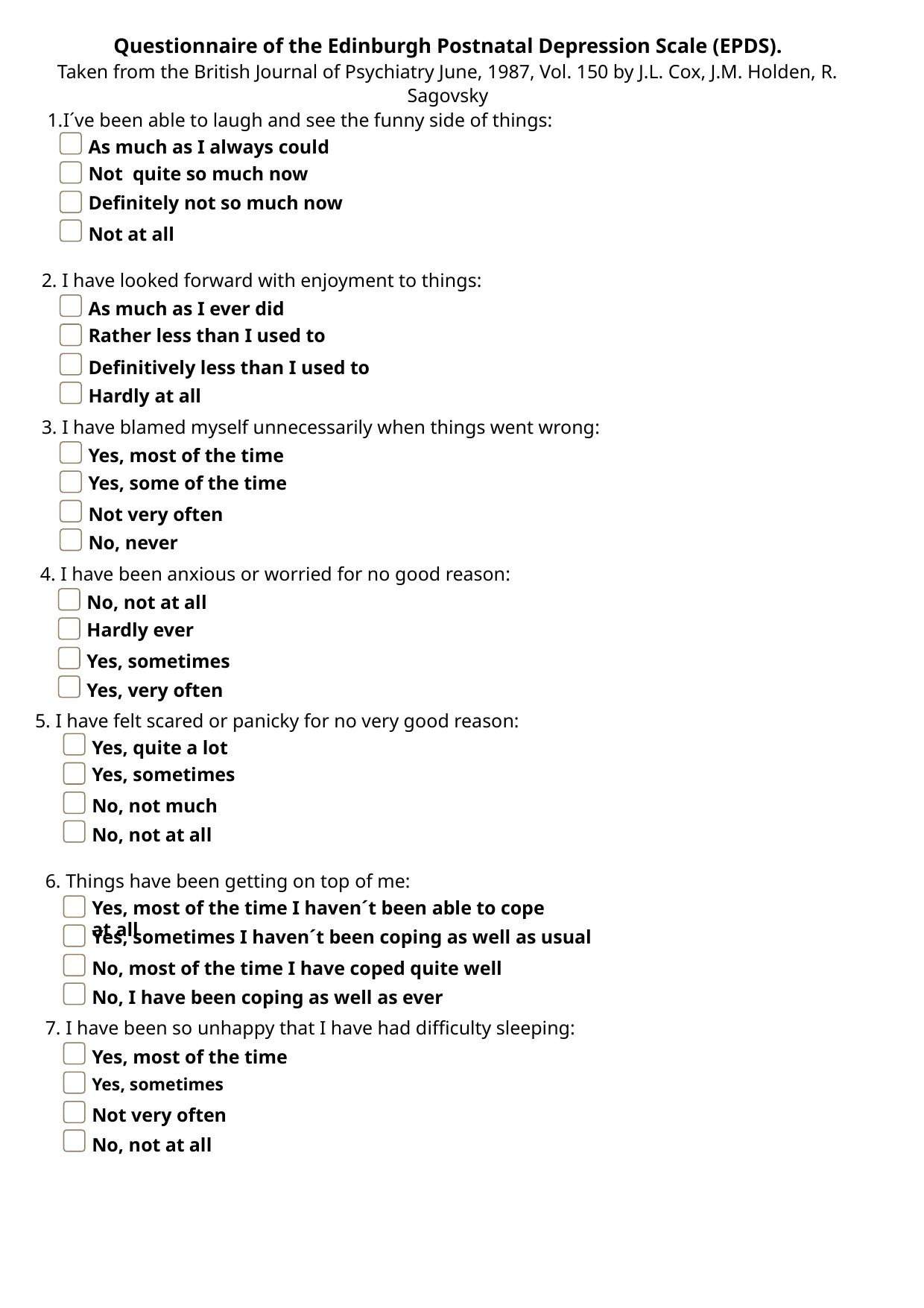

Questionnaire of the Edinburgh Postnatal Depression Scale (EPDS).
Taken from the British Journal of Psychiatry June, 1987, Vol. 150 by J.L. Cox, J.M. Holden, R. Sagovsky
I´ve been able to laugh and see the funny side of things:
As much as I always could
Not quite so much now
Definitely not so much now
Not at all
2. I have looked forward with enjoyment to things:
As much as I ever did
Rather less than I used to
Definitively less than I used to
Hardly at all
3. I have blamed myself unnecessarily when things went wrong:
Yes, most of the time
Yes, some of the time
Not very often
No, never
4. I have been anxious or worried for no good reason:
No, not at all
Hardly ever
Yes, sometimes
Yes, very often
5. I have felt scared or panicky for no very good reason:
Yes, quite a lot
Yes, sometimes
No, not much
No, not at all
6. Things have been getting on top of me:
Yes, most of the time I haven´t been able to cope at all
Yes, sometimes I haven´t been coping as well as usual
No, most of the time I have coped quite well
No, I have been coping as well as ever
7. I have been so unhappy that I have had difficulty sleeping:
Yes, most of the time
Yes, sometimes
Not very often
No, not at all

## Slide 14
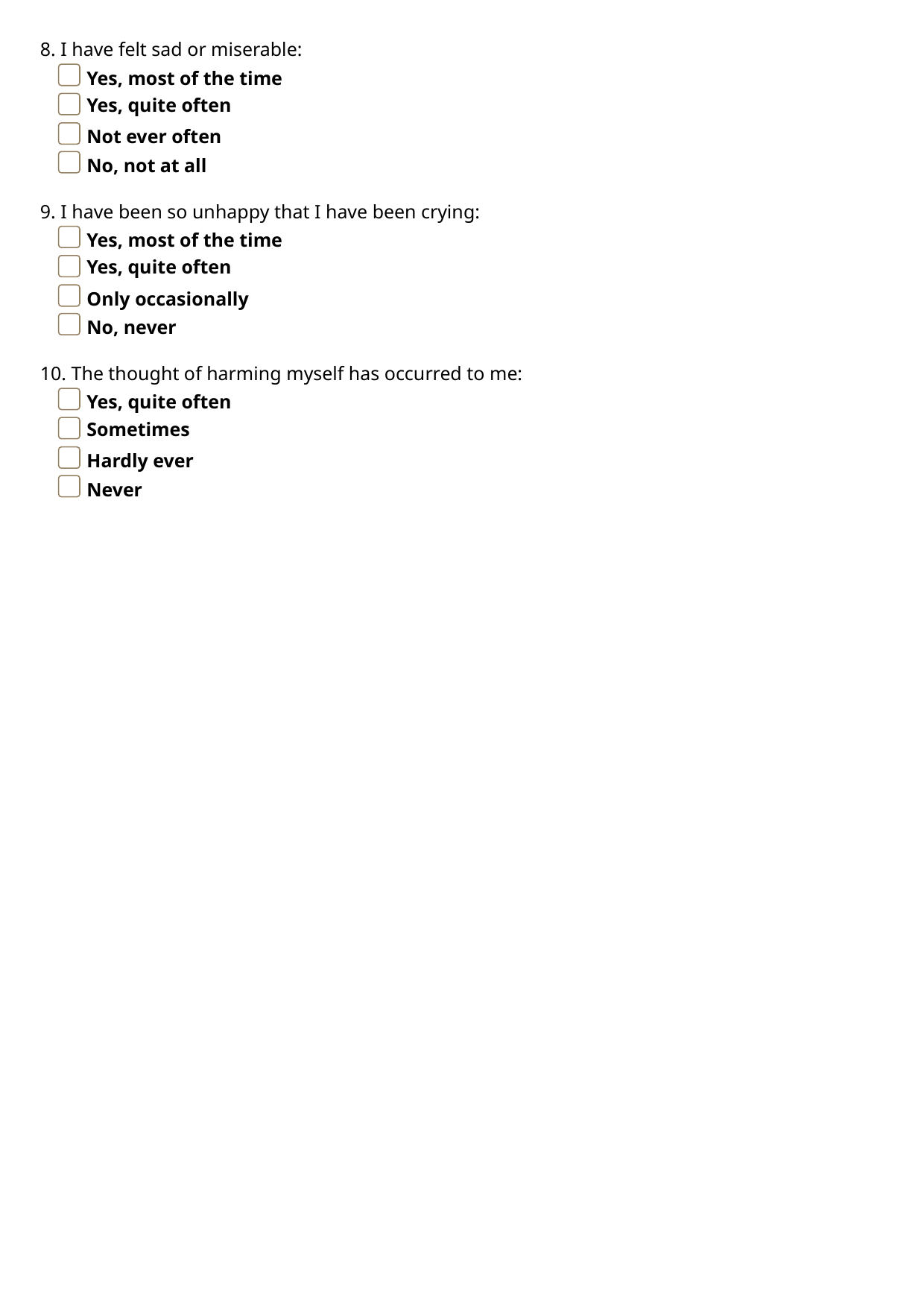

8. I have felt sad or miserable:
Yes, most of the time
Yes, quite often
Not ever often
No, not at all
9. I have been so unhappy that I have been crying:
Yes, most of the time
Yes, quite often
Only occasionally
No, never
10. The thought of harming myself has occurred to me:
Yes, quite often
Sometimes
Hardly ever
Never

## Slide 15
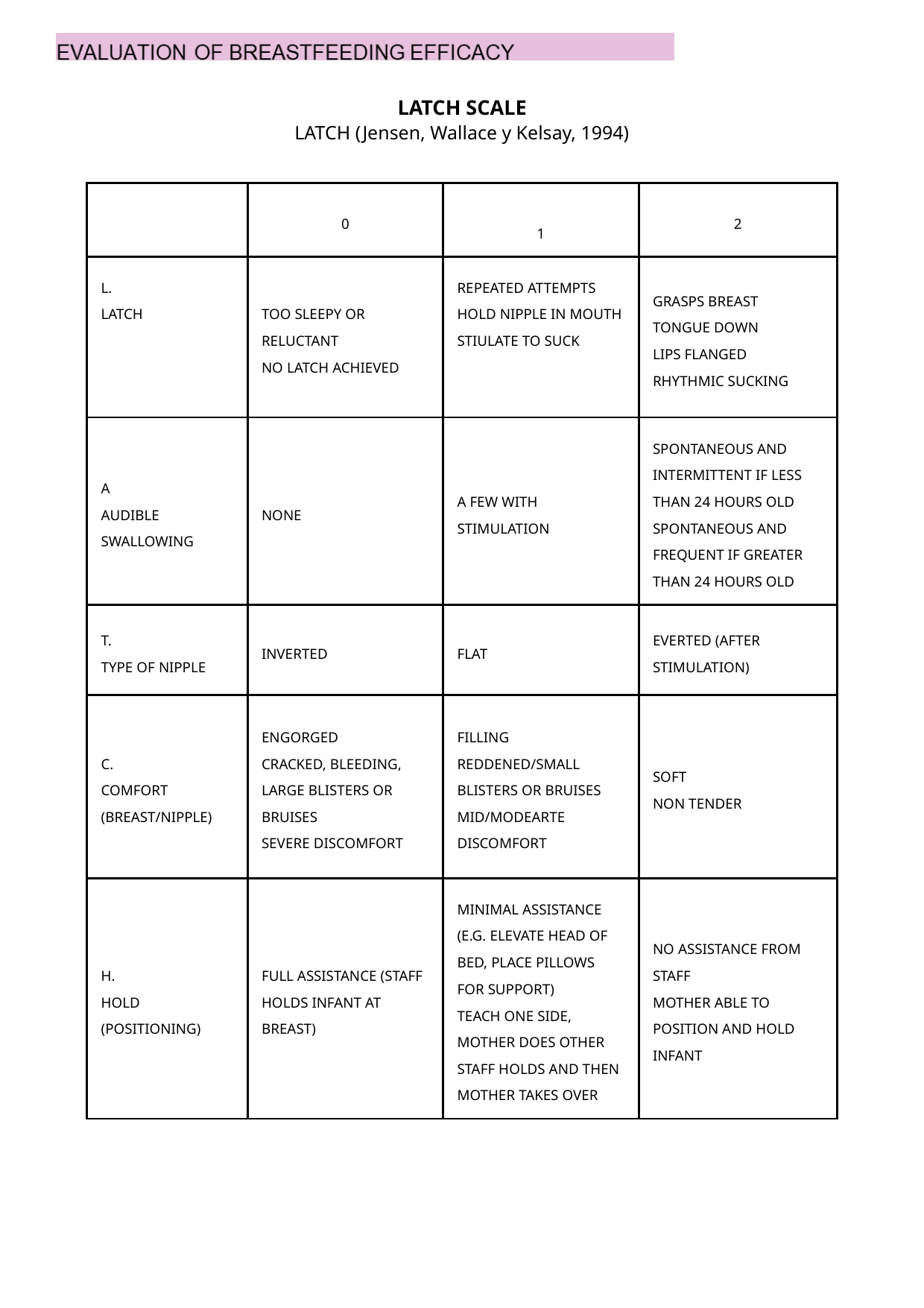

LATCH SCALE
LATCH (Jensen, Wallace y Kelsay, 1994)
| | 0 | 1 | 2 |
| --- | --- | --- | --- |
| L. LATCH | TOO SLEEPY OR RELUCTANT NO LATCH ACHIEVED | REPEATED ATTEMPTS HOLD NIPPLE IN MOUTH STIULATE TO SUCK | GRASPS BREAST TONGUE DOWN LIPS FLANGED RHYTHMIC SUCKING |
| A AUDIBLE SWALLOWING | NONE | A FEW WITH STIMULATION | SPONTANEOUS AND INTERMITTENT IF LESS THAN 24 HOURS OLD SPONTANEOUS AND FREQUENT IF GREATER THAN 24 HOURS OLD |
| T. TYPE OF NIPPLE | INVERTED | FLAT | EVERTED (AFTER STIMULATION) |
| C. COMFORT (BREAST/NIPPLE) | ENGORGED CRACKED, BLEEDING, LARGE BLISTERS OR BRUISES SEVERE DISCOMFORT | FILLING REDDENED/SMALL BLISTERS OR BRUISES MID/MODEARTE DISCOMFORT | SOFT NON TENDER |
| H. HOLD (POSITIONING) | FULL ASSISTANCE (STAFF HOLDS INFANT AT BREAST) | MINIMAL ASSISTANCE (E.G. ELEVATE HEAD OF BED, PLACE PILLOWS FOR SUPPORT) TEACH ONE SIDE, MOTHER DOES OTHER STAFF HOLDS AND THEN MOTHER TAKES OVER | NO ASSISTANCE FROM STAFF MOTHER ABLE TO POSITION AND HOLD INFANT |

## Slide 16
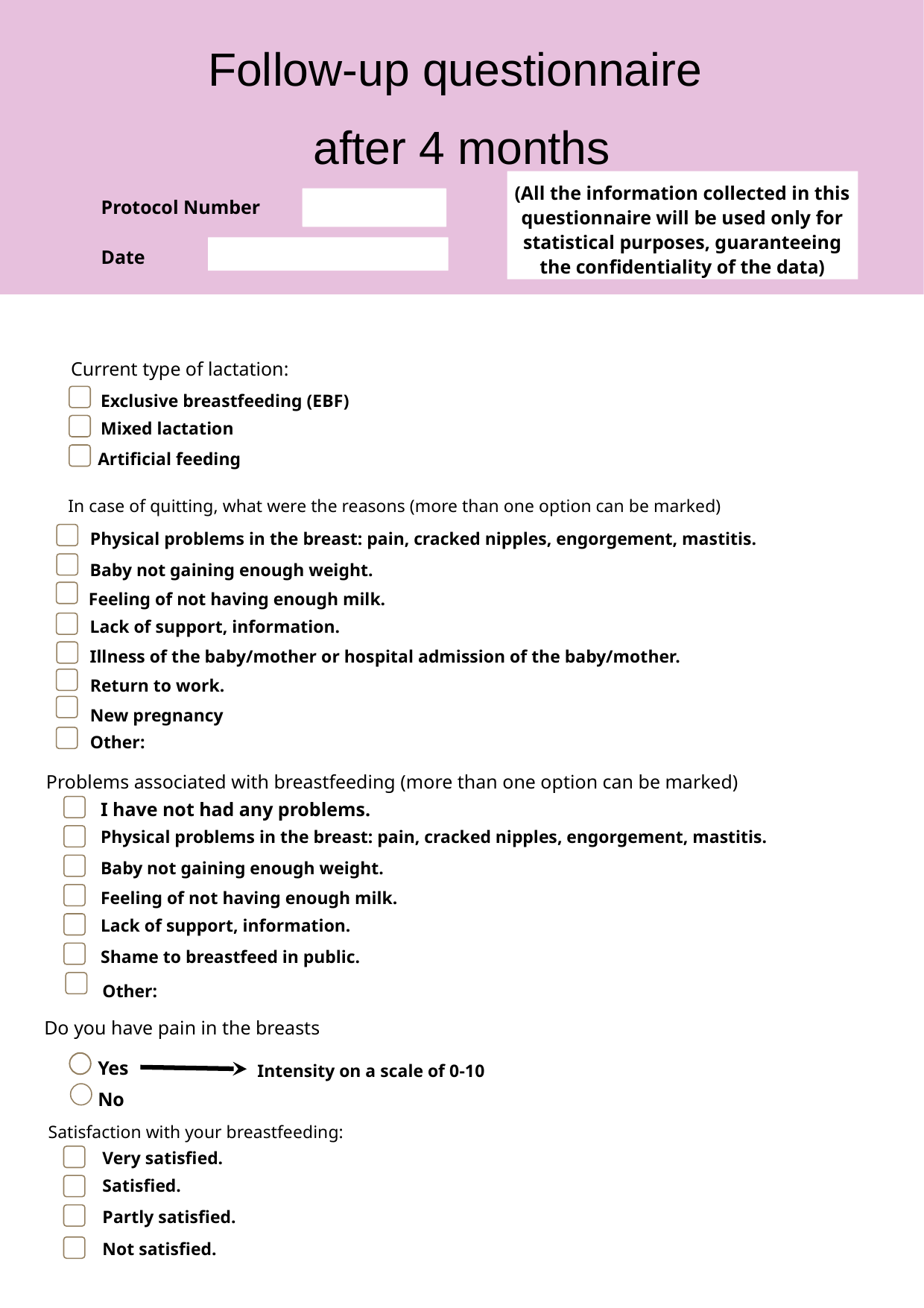

Follow-up questionnaire
after 4 months
(All the information collected in this questionnaire will be used only for statistical purposes, guaranteeing the confidentiality of the data)
Protocol Number
Date
Current type of lactation:
Exclusive breastfeeding (EBF)
Mixed lactation
Artificial feeding
In case of quitting, what were the reasons (more than one option can be marked)
Physical problems in the breast: pain, cracked nipples, engorgement, mastitis.
Baby not gaining enough weight.
Feeling of not having enough milk.
Lack of support, information.
Illness of the baby/mother or hospital admission of the baby/mother.
Return to work.
New pregnancy
Other:
Problems associated with breastfeeding (more than one option can be marked)
I have not had any problems.
Physical problems in the breast: pain, cracked nipples, engorgement, mastitis.
Baby not gaining enough weight.
Feeling of not having enough milk.
Lack of support, information.
Shame to breastfeed in public.
Other:
Do you have pain in the breasts
Yes
Intensity on a scale of 0-10
No
Satisfaction with your breastfeeding:
Very satisfied.
Satisfied.
Partly satisfied.
Not satisfied.

## Slide 17
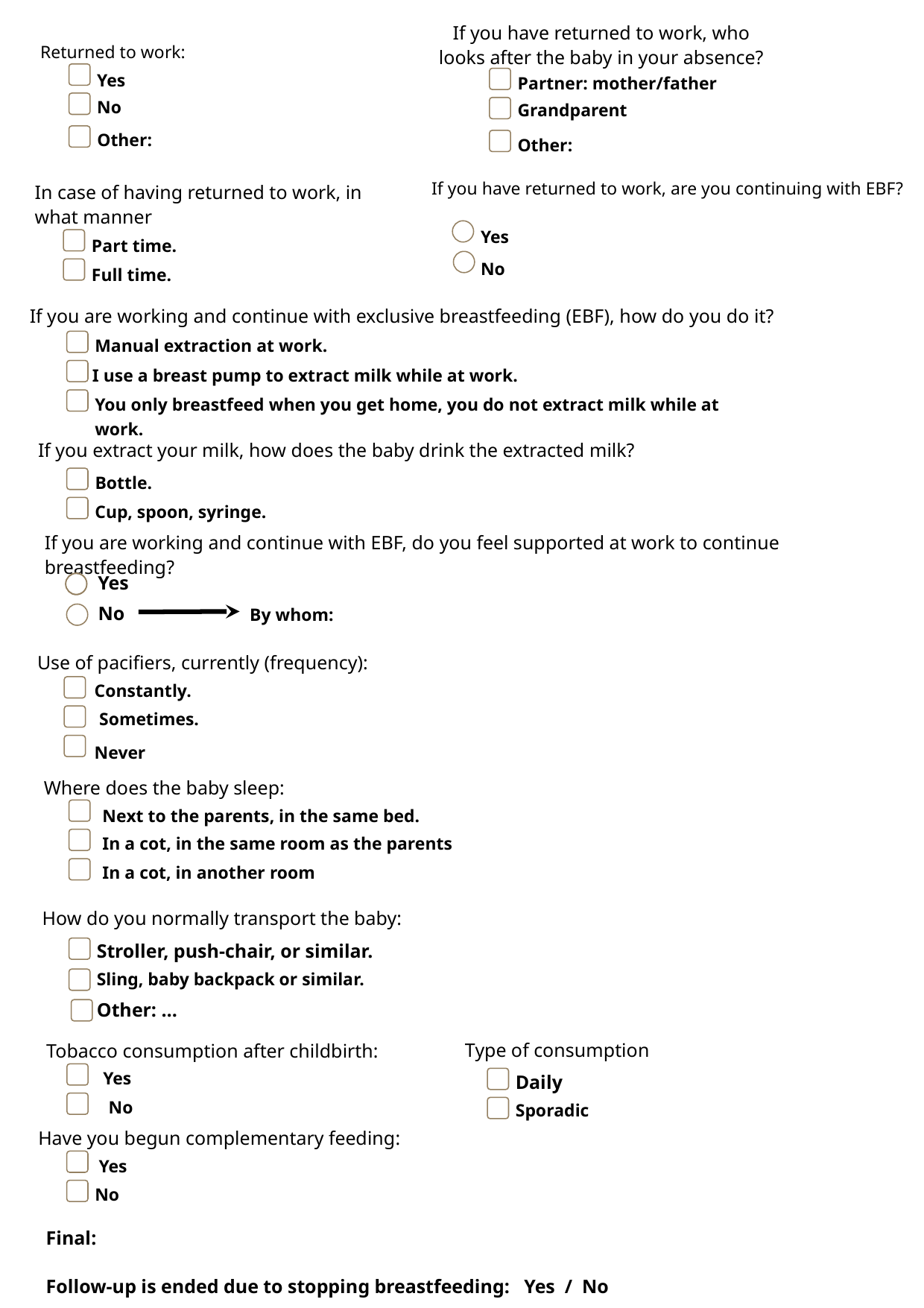

If you have returned to work, who looks after the baby in your absence?
Returned to work:
Yes
Partner: mother/father
No
Grandparent
Other:
Other:
If you have returned to work, are you continuing with EBF?
In case of having returned to work, in what manner
Yes
Part time.
No
Full time.
If you are working and continue with exclusive breastfeeding (EBF), how do you do it?
Manual extraction at work.
I use a breast pump to extract milk while at work.
You only breastfeed when you get home, you do not extract milk while at work.
If you extract your milk, how does the baby drink the extracted milk?
Bottle.
Cup, spoon, syringe.
If you are working and continue with EBF, do you feel supported at work to continue breastfeeding?
Yes
No
By whom:
Use of pacifiers, currently (frequency):
Constantly.
Sometimes.
Never
 Where does the baby sleep:
Next to the parents, in the same bed.
In a cot, in the same room as the parents
In a cot, in another room
How do you normally transport the baby:
Stroller, push-chair, or similar.
Sling, baby backpack or similar.
Other: …
Type of consumption
Tobacco consumption after childbirth:
Yes
Daily
No
Sporadic
Have you begun complementary feeding:
Yes
No
Final:
Follow-up is ended due to stopping breastfeeding: Yes / No

## Slide 18
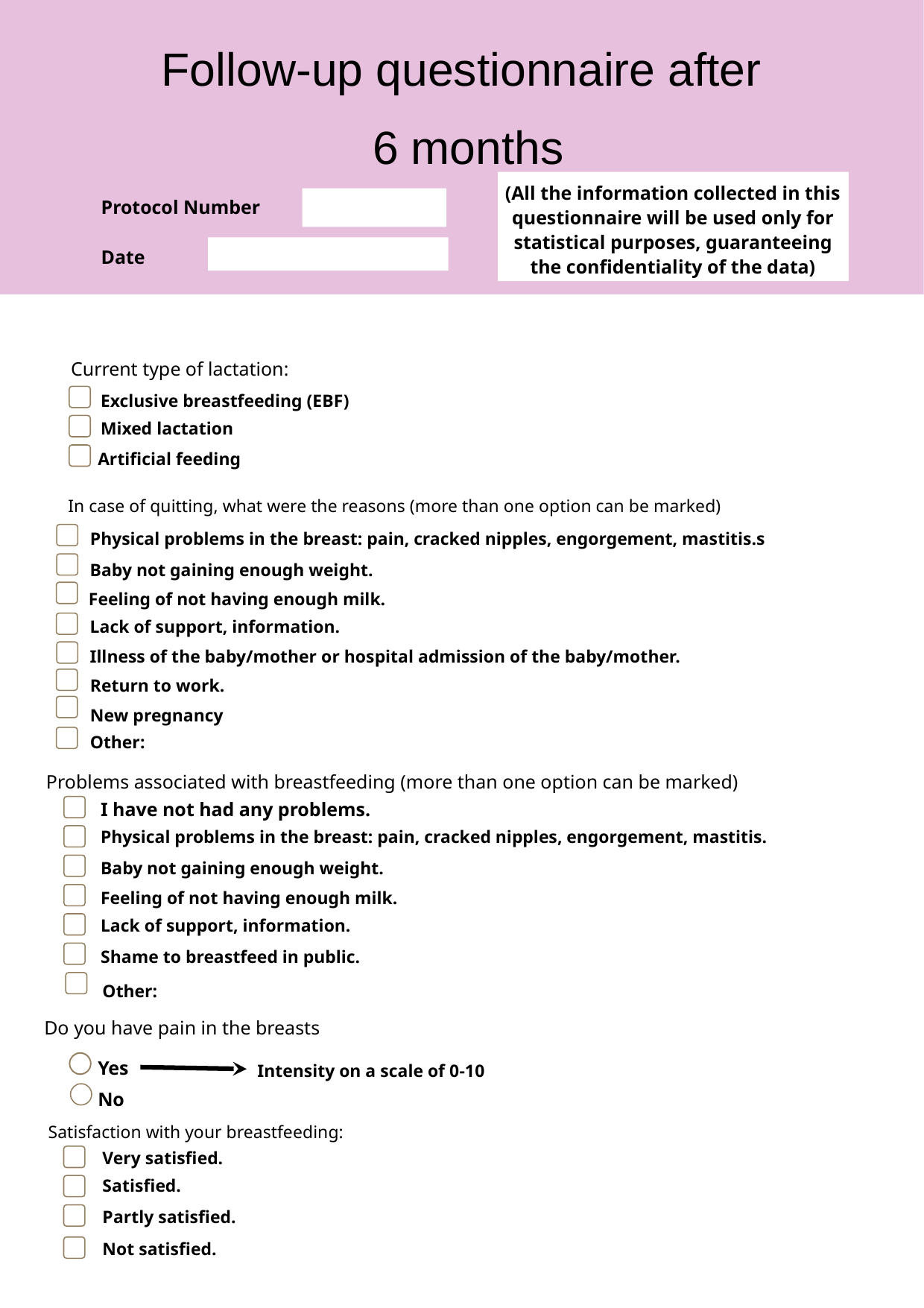

Follow-up questionnaire after
 6 months
(All the information collected in this questionnaire will be used only for statistical purposes, guaranteeing the confidentiality of the data)
Protocol Number
Date
Current type of lactation:
Exclusive breastfeeding (EBF)
Mixed lactation
Artificial feeding
In case of quitting, what were the reasons (more than one option can be marked)
Physical problems in the breast: pain, cracked nipples, engorgement, mastitis.s
Baby not gaining enough weight.
Feeling of not having enough milk.
Lack of support, information.
Illness of the baby/mother or hospital admission of the baby/mother.
Return to work.
New pregnancy
Other:
Problems associated with breastfeeding (more than one option can be marked)
I have not had any problems.
Physical problems in the breast: pain, cracked nipples, engorgement, mastitis.
Baby not gaining enough weight.
Feeling of not having enough milk.
Lack of support, information.
Shame to breastfeed in public.
Other:
Do you have pain in the breasts
Yes
Intensity on a scale of 0-10
No
Satisfaction with your breastfeeding:
Very satisfied.
Satisfied.
Partly satisfied.
Not satisfied.

## Slide 19
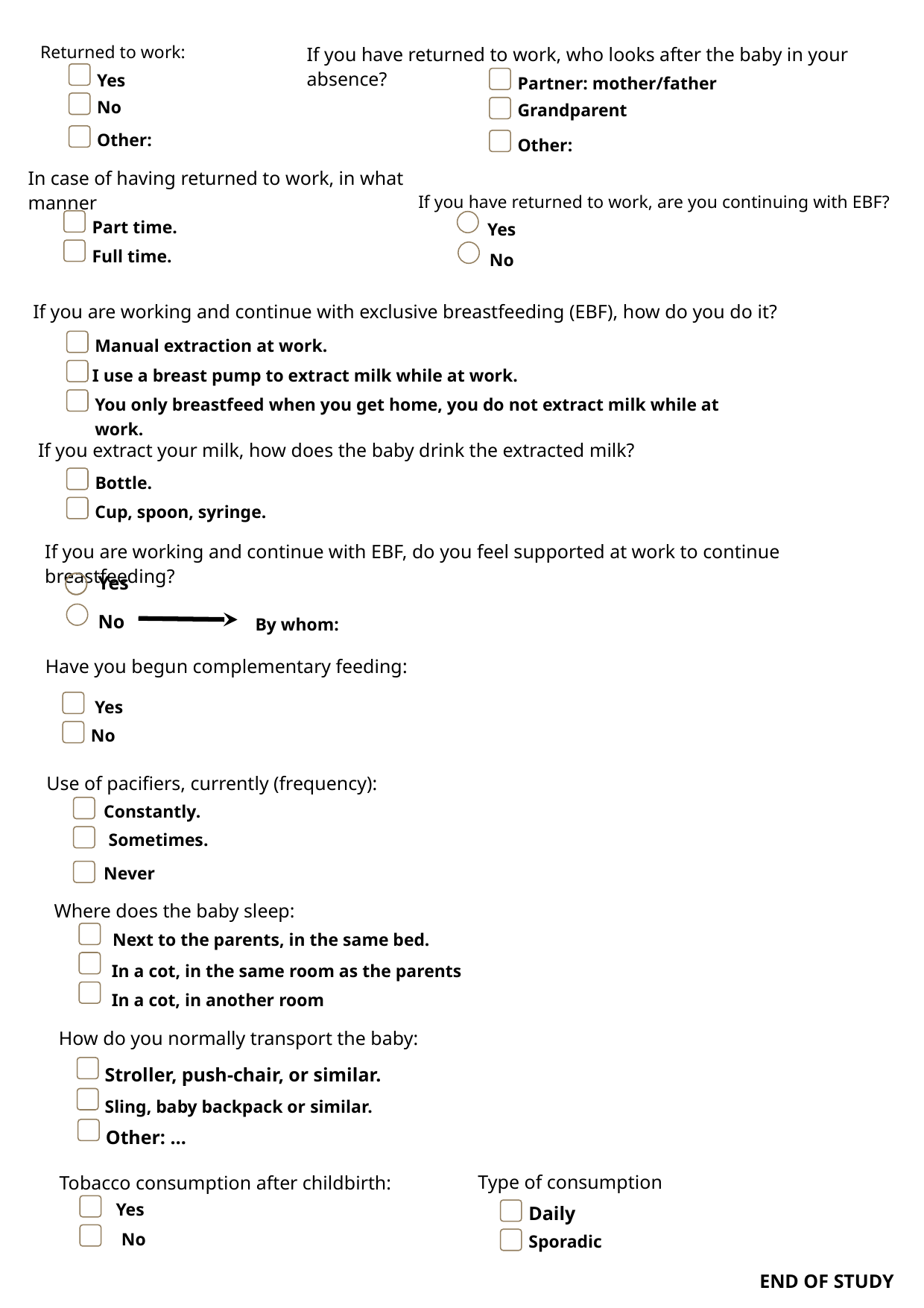

Returned to work:
If you have returned to work, who looks after the baby in your absence?
Yes
Partner: mother/father
No
Grandparent
Other:
Other:
In case of having returned to work, in what manner
If you have returned to work, are you continuing with EBF?
Part time.
Yes
Full time.
No
If you are working and continue with exclusive breastfeeding (EBF), how do you do it?
Manual extraction at work.
I use a breast pump to extract milk while at work.
You only breastfeed when you get home, you do not extract milk while at work.
If you extract your milk, how does the baby drink the extracted milk?
Bottle.
Cup, spoon, syringe.
If you are working and continue with EBF, do you feel supported at work to continue breastfeeding?
Yes
No
By whom:
Have you begun complementary feeding:
Yes
No
Use of pacifiers, currently (frequency):
Constantly.
Sometimes.
Never
 Where does the baby sleep:
Next to the parents, in the same bed.
In a cot, in the same room as the parents
In a cot, in another room
How do you normally transport the baby:
Stroller, push-chair, or similar.
Sling, baby backpack or similar.
Other: …
Type of consumption
Tobacco consumption after childbirth:
Yes
Daily
No
Sporadic
END OF STUDY
